# Euler’s elastica and curvature based model for image restoration

**DOI:** 10.1371/journal.pone.0202464

**Published:** 2018-09-19

**Authors:** Mushtaq Ahmad Khan, Wen Chen, Asmat Ullah, Lin Ji

**Affiliations:** State Key Laboratory of Hydrology-Water Resources and Hydraulic Engineering, Center for Numerical Simulation Software in Engineering and Sciences, College of Mechanics and Materials, Hohai University, Nanjing, Jiangsu 210098, P. R. China; Beijing University of Technology, CHINA

## Abstract

Minimization functionals related to Euler’s elastica energy has a broad range of applications in computer vision and image processing. This paper proposes a novel Euler’s elastica and curvature-based variational model for image restoration corrupted with multiplicative noise. It combines Euler’s elastica curvature with a Weberized total variation (TV) regularization and gets a novel Euler’s elastica energy and TV-based minimization functional. The combined approach in this variational model can preserve edges while reducing the blocky effect in smooth regions. The implicit gradient descent scheme is applied to efficiently finding the minimizer of the proposed functional. Experimental results demonstrate the effectiveness of the proposed model in visual improvement, as well as an increase in the peak signal-to-noise ratio, compared to the PDE-based methods.

## 1 Introduction

During some phases of the manipulation of an image, some random noise is usually introduced. Therefore, image restoration is the fundamental problem in image processing. Among the restoration models, the variational model has been extremely successful in a wide verity of image restoration problems and remains one of the most active areas of research in mathematical image processing and computer vision. The problem includes additive noise removal and multiplicative noise removal. Multiplicative noise appears in various image processing applications, such as synthetic aperture radar (SAR), medical images, single-particle emission-computed technology, and positron emission tomography. The additive noise problem can be molded as

$$\begin{array}{c} f\left(x,y\right)=g\left(x,y\right)+\eta_{1}\left(x,y\right), \end{array}$$ (1)

where *f* is the observed image, *g* is the original image and *η*_1_ is the additive noise. In literature, many effective numerical methods have been utilized to tackle such models connected with image de-noising having additive noise, for instance, in [1–6]. In the last two decades, variational techniques have been widely studied and investigated for image processing tasks, some of them are related image de-noising. For further details, see [7–14].

Multiplicative noise removal problem can be stated as follow;

$$\begin{array}{c} f\left(x,y\right)=g\left(x,y\right)\eta_{2}\left(x,y\right), \end{array}$$ (2)

where *f* is the observed image, *g* is the original image and *η*_2_ is multiplicative noise. In this direction, researchers have used various total variation-based approaches to solve the multiplicative noise removal problem. The interested reader is referred to [15–20] for more details.

The main advantage of TV-based regularization is that it preserves the edges well, but the images resulting from the application of this technique in the presence of noise lead to piecewise constant function. Thus, the finer details in the original image may not be recovered satisfactorily, and the ramps will give stairs (piecewise constants) even though some efforts have been made to reduce the staircase effect. For further details, see [21–26].

Euler’s elastic was first introduced as a prior curve model by Mumford [27] in computer vision. Then, the Euler’s elastica curvature-based total variation-based regularization was applied to find image inpainting [28, 29], and useful results were obtained in image inpainting and restoration.

In this paper, we develop a new model with an improved regularized term, i.e., Euler’s elastica curvature-based TV regularizer with fourth-order, nonlinear Euler-Lagrange equation. This model restores the images well and substantially reduces the staircase effect while preserving the edges and textures.

The rest of the paper is organized as follows. In section 2, the literature has been reviewed regarding image processing by using TV-based methods and Euler’s elastica and curvature-based energy in image restoration and inpainting models. Section 3, discusses Huang et.al [19] model for the removal of multiplicative noise. The proposed model which is Euler’s elastica and curvature-based total variation model for multiplicative noise removal has been discussed in section 4. Section 5, presents the discretization and numerical implementation of the proposed model. Section 6, describes experimental results to compare the two models regarding the visual quality of restoration (PSNR). Section 7, contains the comparison of our proposed model with two other variational based models for image restoration. The sensitivity of the parameters of the proposed model is explained in Section 8. Section 9, concludes the paper. Finally, the derivation of the proposed Euler-Lagrange PDE has been given in appendix A.

## 2 Background work

In the last few years, many approaches have been used to solve the multiplicative noise removal problem [18, 30, 31]. One of these approaches is the local linear minimum mean square method [32–35], another approach is the anisotropic diffusion method [36–38]; these strategies are based on the statistical information of images and noise, so we have not discussed them in details. Our focus in this paper is on variational methods.

The variational approach is an important paradigm for solving the image de-noising problems when the image is defined in the continuous domain. The total variation is a powerful notion in such variational problems. In recent years, variational methods have got much attention in reducing the multiplicative noise owing to the use of total variation (TV) and nonlocal total variation (NLTV) regularization [10, 39, 40]. In literature, many total variation-based models have been proposed, see [41–46] for more details. The variational-based NLTV models have also been widely applied in image restoration [47, 48]. Steidl and Teuber [48] employed the NLTV as a regularizer to recover multiplicative noise images. Since the NLTV-norm used the relevant image patches and hence gives good qualitative results.

As, in image model, *TV*(*g*) = ∫_*Ω*_|∇*g*|*dx* does not take into account that our visual sensitivity to the regularity or local fluctuation |∇*g*| depends on the ambient intensity level *g* [49]. Since all images are eventually perceived and interpreted by the Human Visual System (HVS); as a result, many researchers have found that the human vision psychology and psychophysics play a significant role in image processing. The classic example is the using of the Just Noticeable Difference Model (JND) in image coding and watermarking techniques [50, 51]. In these fields, the NDJ model is used to control the visual perceptual distortion during the coding procedure and watermark embedding. Weber’s law was first described by Weber [52]. The law reveals the universal influence of the background stimulus *g* on human’s sensitivity to the intensity increment |∇*g*|, or so called JND, in the perception of the both sound and light:

$$\begin{array}{c} \frac{\left|\nabla g\right|}{g}=const. \end{array}$$ (3)

Accoring to Weber’s law [49, 53], when the mean intensity of background is increasing with high value, then the intensity increment ∇*g* also has high value. In literature [19], the authors proposed a nonconvex variational functional of model (2) for multiplicative noise removal:

$$\begin{array}{c} \hat{g}=\underset{g}{\text{min}}\;E\left(g\right)=\int_{\Omega }(\beta_{1}+\frac{\beta_{2}}{g})|\nabla g|dxdy+\int_{\Omega }(log\left(g\right)+\frac{f}{g})dxdy, \end{array}$$ (4)

where the first term is the regularization term and the second term is the nonconvex fitting/fidelity term following the MAP estimator for multiplicative Gama noise. *β*_1_ and *β*_2_ are the two parameters, *f* > 0 in *L*^∞^(*Ω*) is the given data. The first regularization term is TV functional, which preserve important structures such as edges, an important visual cue in human and computer vision. The second term $E_{w}\left(g\right)≔TV\left(logg\right)=\int_{\Omega }\frac{\left|\nabla g\right|}{g}$ is the well known Weberized TV regularization term. To briefly explain the role of this term, we assume that *g* has a gradient ∇*g* ∈ *L*^1^(*Ω*)^2^, then *TV*(*logg*) = ∫_*Ω*_|∇*g*|*dx* and the Weberized local variation is

$$\begin{array}{c} {\left|\nabla g\right|}_{\omega }=\frac{\left|\nabla g\right|}{g}=\frac{1}{g}\frac{\partial g}{\partial \vec{n}},\vec{n}=\frac{\nabla g}{\left|\nabla g\right|}, \end{array}$$ (5)

which encodes the influence of the background intensity according to Weber’s law (3).

Recently many variational models involving higher order derivatives have been widely used in image processing because they reduce the staircase effect during the noise elimination. The use of Euler’s elastic energy minimization model based fourth order derivatives damps out the high-frequency components of images faster than faster than the second order PDE based methods because the associated PDE to the Euler’s elastica minimization is fourth order. So the Euler’s elastica model can reduce the staircase effect, textures and can produce better approximations to the natural image. Indeed, it is also able to preserve the object edges while erasing noise. The Euler’s elastica model is one of the most famous high orders models. It has been successfully applied in various problems image denoising, inpainting, and zooming. In [28] the authors derived are the Euler-Langrage equation, proposed some numerical schemes to solve the corresponding Euler-Language equation and also explained the effect of parameters *α*_1_ and *α*_2_ in Eq (3) in image inpainting.

The Euler’ elastica can be described by using the curvature *κ* of the smooth curve Γ as follows

$$\begin{array}{c} E\left(\Gamma \right)=\int_{\Gamma }(\alpha_{1}+\alpha_{2}{\left|\kappa \right|}^{p}\left(s\right))ds, \end{array}$$ (6)

where *s* is the arc length and *α*_1_ and *α*_2_ are the two positive parameters. In the above functional (6), the first term minimizes the total length while the send term minimizes the power of total curvature, where *p* can be set *p* = 1, 2 in [28, 54]. In this work, we set *p* = 2. The Euler’s elastica of all the level curves of an image *g* can be defined as

$$\begin{array}{c} E=\int_{l=0}^{L}\int_{\gamma l:g=l}(\alpha_{1}+\alpha_{2}{\left|\kappa \right|}^{p}\left(s\right))dsdl, \end{array}$$ (7)

where *γl* represents the level curve with *g* = *l*. The curvature *κ* can be expressed as

$$\begin{array}{c} \kappa =\nabla \cdot \frac{\nabla g}{\left|\nabla g\right|}. \end{array}$$ (8)

Combining the Eqs (7) and (8) and using the co-area formula we get

$$\begin{array}{c} E\left(g\right)=\int_{\Omega }(\alpha_{1}+\alpha_{2}{\left|\kappa \right|}^{p})\left|\nabla g\right|. \end{array}$$ (9)

For image restoration applications, the Euler’s elastica energy (9) can be used as a regularization term.

The Elastica model [28], minimizing the Euler’s elastica energy for image inpainting problem is proposed in the following minimization functional

$$\begin{array}{c} \min_{g}\{J\left(g\right)=\int_{\Omega }(\alpha_{1}+\alpha_{2}{\left|\kappa \right|}^{p})\left|\nabla g\right|dxdy+\frac{\lambda }{2}\int_{\Omega /D}{\left(g-f\right)}^{2}dxdy\}, \end{array}$$ (10)

where *α*_1_ and *α*_2_ are arbitrary positive constants, λ > 0 is a penalty parameter, *p* = 2 is usually chosen, *g* = *g*(*x*, *y*) is the true image to be restored and $\kappa =\nabla \cdot \frac{\nabla g}{\left|\nabla g\right|}$ is the curvature. The virtue of Eq (10) is that the regularization using the Euler’s elastica energy penalizes the integral of the square of the curvature along edges instead of only penalizing the length of the edges as the TV model does (if taking *α*_2_ = 0) [10]. Motivated by the applications of Euler’s elastica in image inpainting and restoration and Weberized TV- regularization in image restoration, we propose a new model based on the theory of Euler’s elastica energy and Weberized TV-regularization for multiplicative noise removal problem.

## 3 Li-Li Huang model (M1)

Li-Li Huang et al. proposed the non-convex multiplicative noise removal model using the total variation (TV) filter in [19]. This model achieved some useful restoration results.

The minimization functional by this approach for model (2) is given in (4) which is provided as follow;

$$\begin{array}{c} \hat{g}=\min_{g}E\left(g\right)=\int_{\Omega }(\beta_{1}+\frac{\beta_{2}}{g})|\nabla g|dxdy+\int_{\Omega }(log\left(g\right)+\frac{f}{g})dxdy. \end{array}$$ (11)

Here, *f* > 0 in *L*^∞^(*Ω*) is the given data in the model. In (11) the first term is the TV-functional which preserve the important structures such as edges, an important cue in the human and computer vision and its second term

$$\begin{array}{c} E_{\omega }\left(g\right)=TV(log(g))=\int_{\Omega }\frac{\left|\nabla g\right|}{g} \end{array}$$ (12)

is called Weberized TV regularization term [53]. We suppose that *g* has the gradient ∇*g* ∈ *L*^1^(*Ω*)^2^, then the above Eq (12) can be re-written as

$$\begin{array}{c} TV(log(g))=\int_{\Omega }\frac{\left|\nabla g\right|}{g}=\int_{\Omega }\frac{\left|\nabla g\right|}{g}dx, \end{array}$$ (13)

with

$$\begin{array}{c} {\left|\nabla g\right|}_{\omega }=\frac{\left|\nabla g\right|}{g}=\frac{1}{g}\frac{\partial g}{\partial \vec{n}},\vec{n}=\frac{\nabla g}{\left|\nabla g\right|}, \end{array}$$ (14)

is the Weberized local variation, which encodes the influence of backward intensity according to Weber’s law [53]. The corresponding Euler-Lagrange of the minimization functional (11) can be defined as

$$\begin{array}{c} -(\frac{\beta_{1}g+\beta_{2}}{g})\nabla \cdot (\frac{\nabla g}{\sqrt{{\left|\nabla g\right|}^{2}+\varepsilon }})+(\frac{g-f}{g^{2}})=0\;in\;\Omega .\;\frac{\partial g}{\partial \vec{n}}=0\;on\;the\;\partial \Omega . \end{array}$$ (15)

Since *g* > 0, so the above Eq (15) can be re-defined as

$$\begin{array}{c} -\nabla \cdot (\frac{\nabla g}{\sqrt{{\left|\nabla g\right|}^{2}+\varepsilon }})+\frac{g-f}{g(\beta_{1}g+\beta_{2})}=0\;in\;\Omega .\;\frac{\partial g}{\partial \vec{n}}=0\;on\;\partial \Omega , \end{array}$$ (16)

or

$$\begin{array}{c} -div(\frac{\nabla g}{\sqrt{{\left|\nabla g\right|}^{2}+\varepsilon }})+\frac{g-f}{g(\beta_{1}g+\beta_{2})}=0\;in\;\Omega .\;\frac{\partial g}{\partial \vec{n}}=0\;on\;\partial \Omega . \end{array}$$ (17)

Define $\tilde{\lambda }=\tilde{\lambda }\left(g\right)=\frac{1}{g(\beta_{1}g+\beta_{2})}$, then Eq (17) implies

$$\begin{array}{c} \nabla E\left(g\right)=-div(\frac{\nabla g}{\sqrt{{\left|\nabla g\right|}^{2}+\varepsilon }})+\tilde{\lambda }(g-f)=0\;in\;\Omega , \end{array}$$ (18)

with Neumann boundary conditions. Additive operator splitting (AOS) method [19, 55] has been utilized to solve (18).

## 4 The proposed model (M2)

In this section, we aim to introduce a new model using both Euler’s elastica curvature energy and Weberized total variation (TV)-norm as a regularizer. This model apparently uses the advantages of both Euler’s elastica curvature energy and Weberized TV-norm, which leads to good restoration results. In this model, the Euler’s elastica energy, denoised images have smooth connections in the level curves of images which lead to good performance regarding image restoration (PSNR), elimination of staircase effect, and preservation of textures, while the Weberized total variation preserves jump discontinuities and sharp the edges in images. This is believed to be better estimation to a natural image than a piecewise constant approximation in the smooth regions. Hence using this model, consistent improvement in PSNR values is obtained.

As the minimization approach for model (2) by Huang et al. [19] is defined as

$$\begin{array}{c} \hat{g}=\underset{g}{\text{min}}\;E\left(g\right)=\int_{\Omega }(\beta_{1}+\frac{\beta_{2}}{g})R\left(g\right)dxdy+\int_{\Omega }(log\left(g\right)+\frac{f}{g})dxdy, \end{array}$$ (19)

where *R*(*g*) = |∇*g*|.

But Euler’s elastica and curvature-based inpainting model [28] is given by the equation.

$$\begin{array}{c} \underset{g}{\text{min}}\{J\left(g\right)=\int_{\Omega }R\left(g\right)dxdy+\frac{\lambda }{2}\int_{\Omega /D}(g-f)dxdy\}, \end{array}$$ (20)

having

$$\begin{array}{c} R\left(g\right)=(\alpha_{1}+\alpha_{2}\kappa^{2})\left|\nabla g\right|, \end{array}$$ (21)

where *α*_1_ and *α*_2_ are the parameters and *κ* is the curvature and is defined as follows.

$$\begin{array}{c} \kappa =\kappa (x,y)=\nabla \cdot \frac{\nabla g}{\left|\nabla g\right|}. \end{array}$$ (22)

Combining Eqs (19) and (21), the following equation is obtained.

$$\begin{array}{c} \hat{g}=\underset{g}{\text{min}}\;E\left(g\right)=\int_{\Omega }(\beta_{1}+\frac{\beta_{2}}{g})(\alpha_{1}+\alpha_{2}\kappa^{2})\left|\nabla g\right|dxdy+\int_{\Omega }(log\left(g\right)+\frac{f}{g})dxdy. \end{array}$$ (23)

Hence, (23) is a new high-order curvature-based total variational minimization functional for multiplicative noise removal problem. The first term is called regularization term and the second term is called non-convex data fitting term, where *β*_1_, *β*_2_, *α*_1_, and *α*_2_ are the four regularization parameters which usually depends on the noise level and type of the image.

The corresponding Euler-Lagrange equation of (23) is given as

$$\begin{array}{c} -(\frac{\beta_{1}g+\beta_{2}}{g})(\nabla \cdot U)+(\frac{g-f}{g^{2}})=0, \end{array}$$ (24)

or

$$\begin{array}{c} -(\nabla \cdot U)+\frac{1}{g(\beta_{1}u+\beta_{2})}(g-f)=0, \end{array}$$ (25)

or

$$\begin{array}{c} -(\nabla \cdot U)+\tilde{\lambda }(g-f)=0, \end{array}$$ (26)

where

$$\begin{array}{c} \tilde{\lambda }=\tilde{\lambda }\left(g\right)=\frac{1}{g(\beta_{1}g+\beta_{2})}, \end{array}$$ (27)

and

$$\begin{array}{c} U=[(\alpha_{1}+\alpha_{2}\kappa^{2})\cdot \frac{\nabla g}{\left|\nabla g\right|}-\frac{2\alpha_{2}}{|\nabla g{|}^{3}}\nabla^{⊥}g\nabla (\kappa |\nabla g|)\nabla^{⊥}g], \end{array}$$ (28)

with boundary conditions $\frac{\partial g}{\partial n}=0$ and $\frac{\partial (\alpha_{1}+\alpha_{2}\kappa^{2})}{\partial n}=0$. Where ∇*g* in (28) is the normal vector and ∇^⊥^*g* is the corresponding tangent vector i.e

$$\begin{array}{c} \vec{n}=\nabla g=(g_{x},g_{y}),\;t=\nabla^{⊥}g=(-g_{y},g_{x}). \end{array}$$ (29)

From Eq (26) *U* = (*U*^1^, *U*^2^), where

$$\begin{array}{c} U^{1}=(\alpha_{1}+\alpha_{2}\kappa^{2})\frac{\partial_{x}g}{\left|\nabla g\right|}-\frac{2\alpha_{2}}{|\nabla g{|}^{3}}[-\partial_{y}g\partial_{x}(\kappa |\nabla g|)+\partial_{x}g\partial_{y}(\kappa |\nabla g|)]\partial_{y}g, \end{array}$$ (30)

and

$$\begin{array}{c} U^{2}=(\alpha_{1}+\alpha_{2}\kappa^{2})\frac{\partial_{y}g}{\left|\nabla g\right|}-\frac{2\alpha_{2}}{|\nabla g{|}^{3}}[-\partial_{y}g\partial_{x}(\kappa |\nabla g|)+\partial_{x}g\partial_{y}(\kappa |\nabla g|)]\partial_{x}g. \end{array}$$ (31)

## 5 Numerical implementation

To discretize the Eq (26), which is a fourth-order PDE, we use the finite difference method discussed in [56–58]. Here, we include the details for the sake of the completeness of the present discussion.

In this discussion, we use the notation *u*_(*i*, *j*)_ = *u*(*i*Δ*x*, *j*Δ*y*), where *i*, *j* are integers, Δ*x*, Δ*y* are the space step sizes along the *x* and *y* directions, respectively; and (*i*Δ*x*, *j*Δ*y*) represents a discrete point.

Here

$$\begin{array}{c} \nabla \cdot U=\frac{\partial }{\partial x}U^{1}+\frac{\partial }{\partial y}U^{2}. \end{array}$$ (32)

At(*i*, *j*) we can write Eq (32)

$$\begin{array}{c} \nabla \cdot U_{i,j}=(\frac{U_{i+\frac{1}{2},j}^{1}-U_{i-\frac{1}{2},j}^{1}}{\Delta x})+(\frac{U_{i,j+\frac{1}{2}}^{2}-U_{i,j-\frac{1}{2}}^{2}}{\Delta y}). \end{array}$$ (33)

Using the Eq (33), we approximate $U_{i+\frac{1}{2},j}^{1}$ and $U_{i-\frac{1}{2},j}^{1}$ as follow;

*Curvature term*. These are approximated by min-mode of two adjacent whole pixels.

$$\begin{array}{c} \kappa_{i+\frac{1}{2},j}=min-mod(\kappa_{i+1,j},\kappa_{i,j}),\kappa_{i-\frac{1}{2},j}=min-mod(\kappa_{i,j},\kappa_{i-1,j}), \end{array}$$ (34)

where

$$\begin{array}{c} min-mod(m,n)=(\frac{sgnm+sgnn}{2})min(|m|,|n|). \end{array}$$ (35)

*Partial derivatives in x*. By the central difference of two adjacent whole

$$\begin{array}{c} \partial x(g_{i+\frac{1}{2},j})=\frac{1}{\Delta x}(g_{i+1,j}-g_{i,j}),\;\partial x(g_{i-\frac{1}{2},j})=\frac{1}{\Delta x}(g_{i,j}-g_{i-1,j}), \end{array}$$ (36)

$$\begin{array}{c} g_{x}=\frac{g_{i+1,j}-g_{i,j}}{\Delta x}, \end{array}$$ (37)

$$\partial x{\left(\kappa \left|\nabla g\right|\right)}_{i+\frac{1}{2},j}=\frac{1}{\Delta x}\left(\kappa_{i+1,j}{\left|\nabla g\right|}_{i+1,j}-\kappa_{i,j}{\left|\nabla g\right|}_{i,j}\right)$$ (38)

$$\begin{array}{c} \partial x{\left(\kappa \left|\nabla g\right|\right)}_{i-\frac{1}{2},j}=\frac{1}{\Delta x}\left(\kappa_{i,j}{\left|\nabla g\right|}_{i,j}-\kappa_{i-1,j}{\left|\nabla g\right|}_{i-1,j}\right). \end{array}$$ (39)

Here

$$\begin{array}{c} {\left|\nabla g\right|}_{i,j}=\frac{1}{2}\sqrt{{\left(g_{i+1,j}-g_{i-1,j}{)}^{2}+(g_{i,j+1}-g_{i,j-1}\right)}^{2}+4\Delta x^{2}\epsilon },where\epsilon >0. \end{array}$$ (40)

*Partial derivatives in y*. By min-mod of ∂*y*, *s* at two adjacent whole points

$$\begin{array}{c} \partial y(g_{i+\frac{1}{2},j})=min-mod(\frac{1}{2\Delta y}(g_{i+1,j+1}-g_{i+1,j-1}),\frac{1}{2\Delta y}(g_{i,j+1}-g_{i,j-1})), \end{array}$$ (41)

$$\begin{array}{c} \partial y(g_{i-\frac{1}{2},j})=min-mod(\frac{1}{2h}(g_{i,j+1}-g_{i,j-1}),\frac{1}{2\Delta y}(g_{i-1,j+1}-g_{i-1,j-1})), \end{array}$$ (42)

$$\begin{array}{c} g_{y}=min-mod[\frac{g_{i+1,j+1}-g_{i+1,j-1}}{2\Delta y},\;\frac{g_{i,j+1}-g_{i,j-1}}{2\Delta y}], \end{array}$$ (43)

$$\begin{array}{c} \partial y{\left(\kappa \left|\nabla g\right|\right)}_{i+\frac{1}{2},j}=min-mod\left(m,n\right), \end{array}$$ (44)

with

$$\begin{array}{c} m=\frac{1}{2}(\kappa_{i,j+1}{\left|\nabla g\right|}_{i,j+1}-\kappa_{i,j-1}{\left|\nabla g\right|}_{i,j-1}), \end{array}$$ (45)

and

$$\begin{array}{c} n=\frac{1}{2}(\kappa_{i-1,j+1}{\left|\nabla g\right|}_{i-1,j+1}-\kappa_{i-1,j-1}{\left|\nabla g\right|}_{i-1,j-1}). \end{array}$$ (46)

Also

$$\begin{array}{c} {\left|\nabla g\right|}_{i+\frac{1}{2},j}=\sqrt{{\left(\partial x(g_{i+\frac{1}{2},j})\right)}^{2}+(\partial y(g_{i,j+\frac{1}{2}}){)}^{2}+\varepsilon }, \end{array}$$ (47)

and

$$\begin{array}{c} {\left|\nabla g\right|}_{i-\frac{1}{2},j}=\sqrt{(\partial x(g_{i-\frac{1}{2},j}){)}^{2}+{\left(\partial y(g_{i,j-\frac{1}{2}})\right)}^{2}+\varepsilon }. \end{array}$$ (48)

By similar way we can find the approximations for $U_{i,j+\frac{1}{2}}^{2}$ and $U_{i,j-\frac{1}{2}}^{2}$ from Eq (33) with Neumann boundary conditions

$$\begin{array}{c} g_{i,0}=g_{i,1},g_{i,n+1}=g_{i,n},g_{0,j}=g_{1,j},g_{m+1,j}=g_{m,j}. \end{array}$$ (49)

As the steady state form of Eq (26) is

$$\begin{array}{c} \frac{\partial g}{\partial t}=(\nabla \cdot U)+\tilde{\lambda }(g-f). \end{array}$$ (50)

At (*i*, *j*), we approximate Eq (50) by using (33) as follow;

$$\begin{array}{c} \frac{g_{\left(i,j\right)}^{n+1}-g_{\left(i,j\right)}^{n}}{dt}=(\frac{U_{i+\frac{1}{2},j}^{1}-U_{i-\frac{1}{2},j}^{1}}{\Delta x})+(\frac{U_{i,j+\frac{1}{2}}^{2}-U_{i,j-\frac{1}{2}}^{2}}{\Delta y})+{\tilde{\lambda }}_{\left(i,j\right)}^{n}(g_{\left(i,j\right)}^{n}-f_{\left(i,j\right)}). \end{array}$$ (51)

Since, implicit gradient descent scheme has achieved good results in [14], so we utilize this scheme to solve the PDE (51), which can be expressed as under;

$$\begin{array}{c} g_{\left(i,j\right)}^{n+1}=g_{\left(i,j\right)}^{n}+dt[(\frac{U_{i+\frac{1}{2},j}^{1}-U_{i-\frac{1}{2},j}^{1}}{\Delta x})+(\frac{U_{i,j+\frac{1}{2}}^{2}-U_{i,j-\frac{1}{2}}^{2}}{\Delta y})+{\tilde{\lambda }}_{\left(i,j\right)}^{n}(g_{\left(i,j\right)}^{n}-f_{\left(i,j\right)})], \end{array}$$ (52)

where $g_{\left(i,j\right)}^{n}$ shows the value of *g* at *ndt* times, *dt* is time step size, and Δ*x* = Δ*y*.

## 6 Numerical experiments

**Fig 1:**
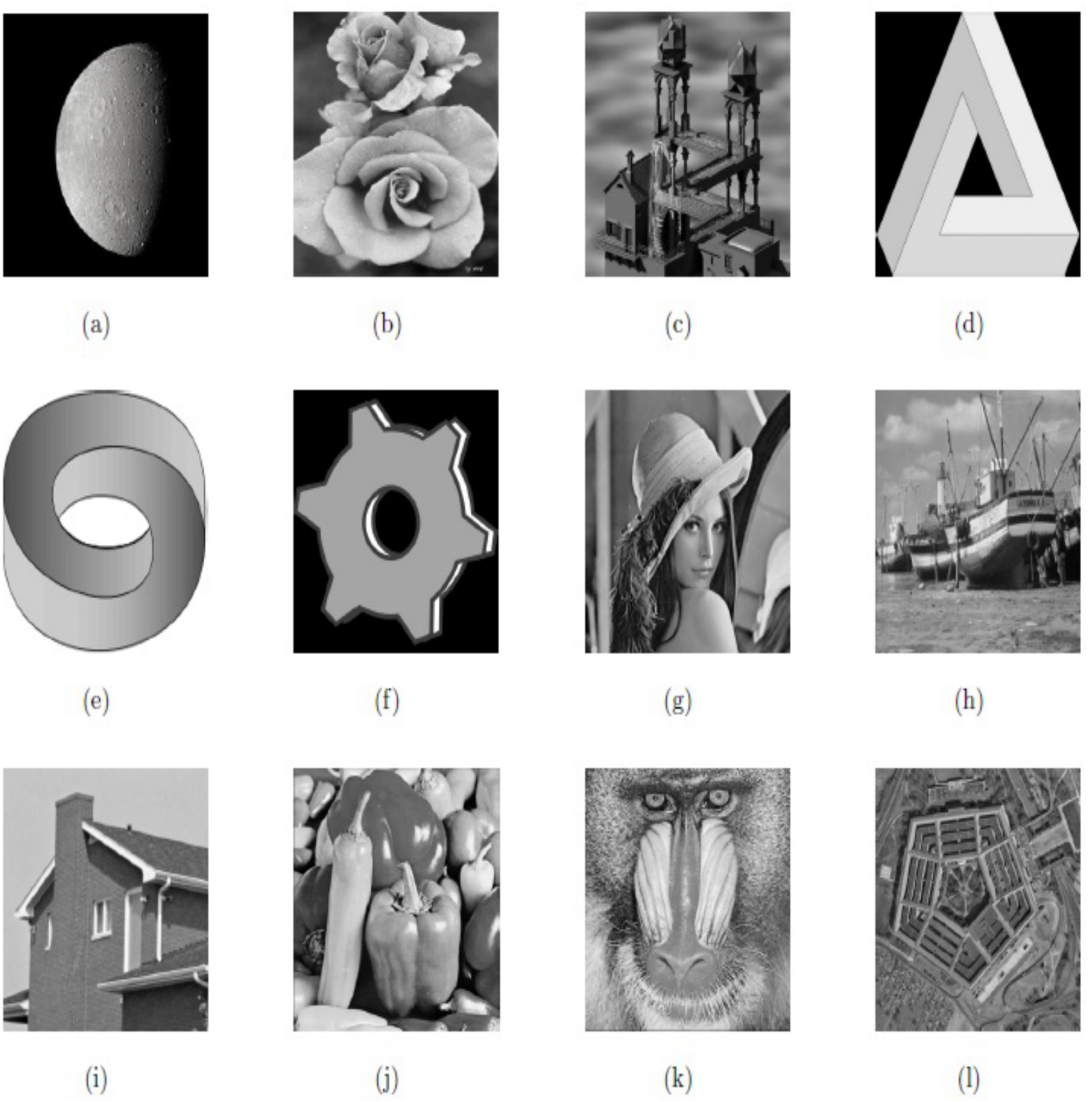
Test images; (a) Moon; (b) Rose; (c) Synthetic1; (d) Synthetic2; (e) Synthetic3; (f) Synthetic4; (g) Lena; (h) Boat; (i) House; (j) Peppers; (k) Baboon; (i) Texture.

In this section, we provide some numerical results from applying our proposed model M2. We also compare them with the results obtained by using the model M1. The test images used for numerical experiments are “Moon”, “Rose”, “Synthetic1”, “Synthetic2”, “Synthetic3”, “Synthetic4” and “Lena”, “Boat”, “House”, “Peppers”, “Baboon”, and “Texture”, respectively, which are shown in Fig 1. We test these images on speckle noise (uniform distribution) with mean value 1 and variance *σ*^2^.

### 6.1 Quality of restoration

Peak signal to noise ratio (PSNR) is used to analyze the quality of the image that has been restored. So here we check the restoration quality of the two models by PSNR as follow:

$$\begin{array}{c} PSNR=10*log_{10}[\frac{M\times Nmax{\left\{\hat{g}\right\}}^{2}}{∥\hat{g}{-g∥}^{2}}], \end{array}$$ (53)

where $\hat{g}$ is the given image, *g* is the restore image and *M* × *N* the size of the image.

**Fig 2:**
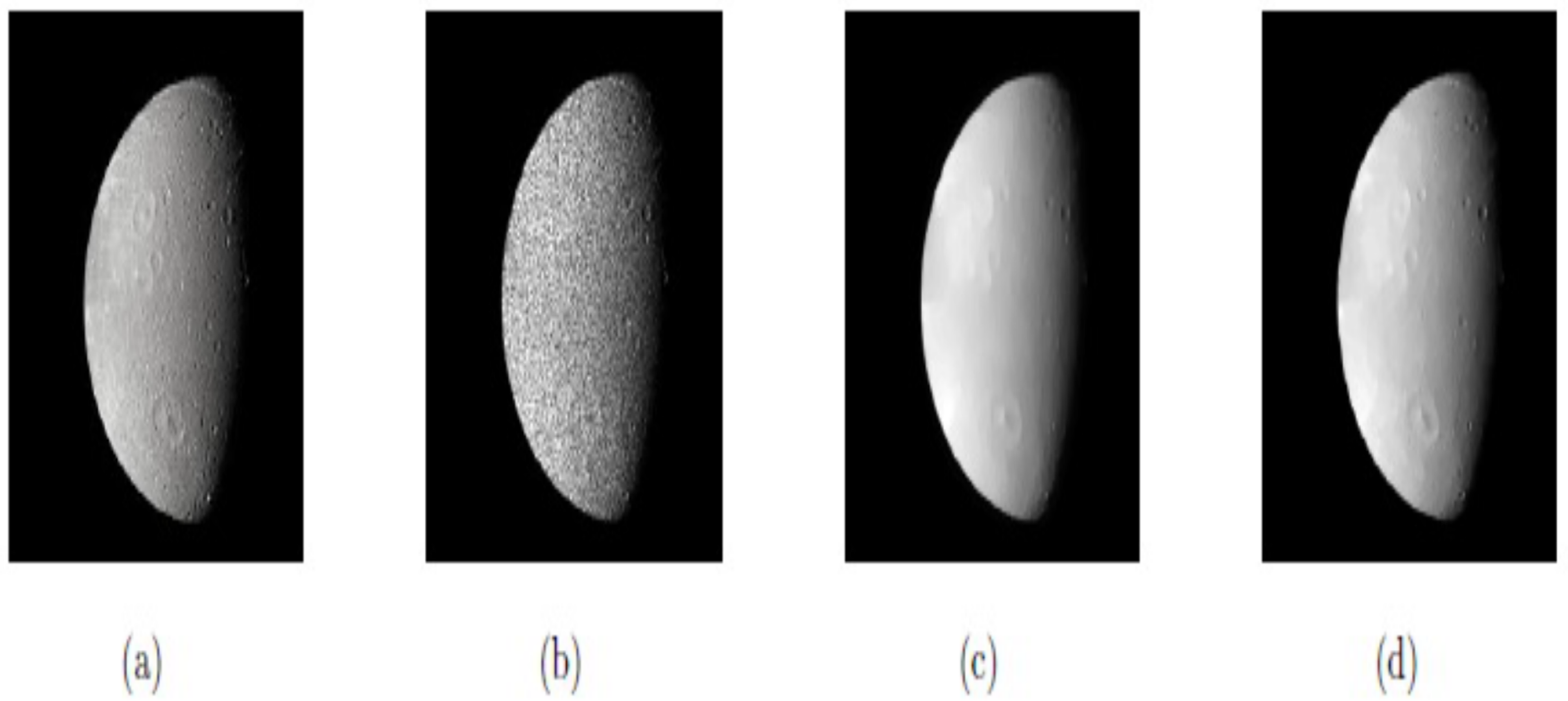
Restored results on real image Moon; (a) Original image; (b) Noisy image with *σ*^2^ = 0.1; (c) Restored image by model M1; (d) Restored image by model M2.

**Fig 3:**
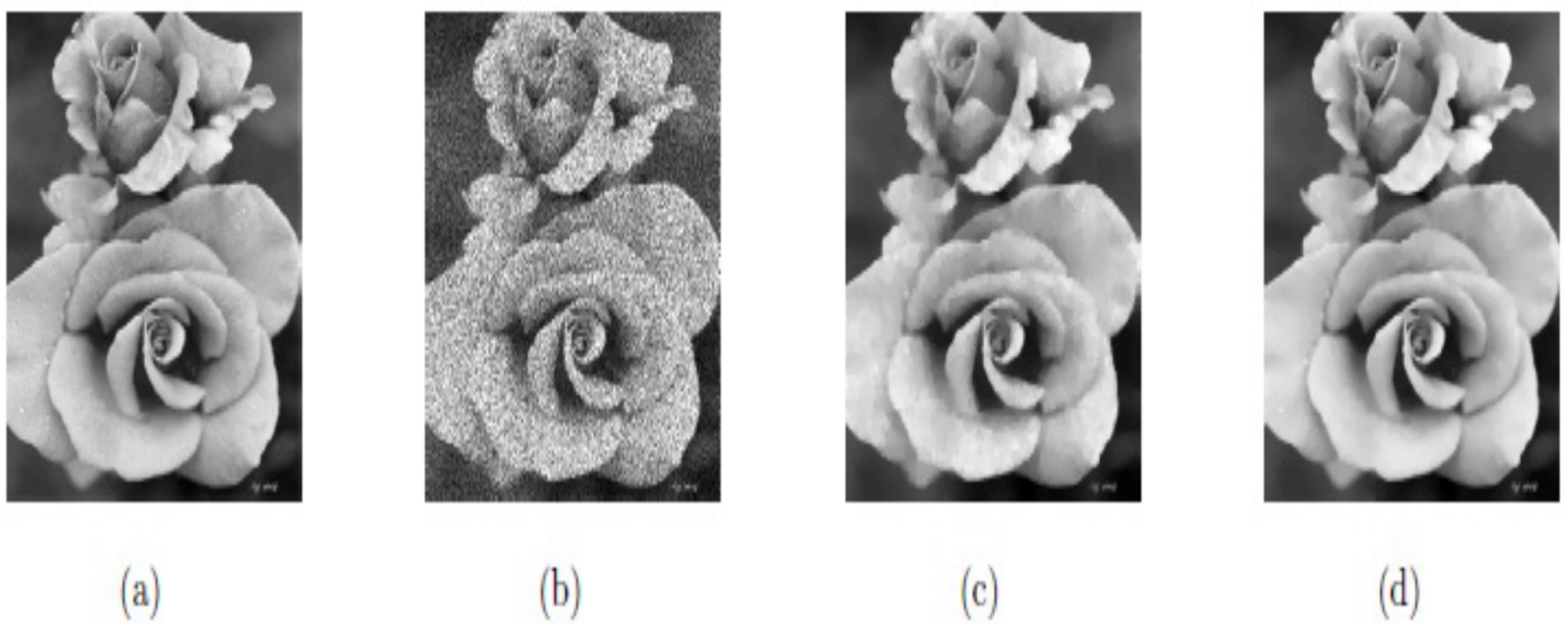
De-noised results on real image Rose; (a) Original image; (b) Noisy image with *σ*^2^ = 0.1; (c) Recovered image by model M1; (d) Recovered image by model M2.

**Example 1**:

In this first example, the two methods M1 and M2 are applied and tested on the natural images “Moon” and “Rose,” having speckle noise with noise variance *σ*^2^ = 0.1, and *σ*^2^ = 0.1, respectively, which are shown in Figs 2 and 3. In both Figs, (a) and (b) are the original and noisy images, while Figs (c) and (d) depict the restored images by methods M1 and M2, respectively. In each case, we can notice that the visual quality of restoration by proposed method M2 is much better than that of method M1. Moreover, the PSNR values for the two images “Moon” and “Rose” for methods M1 and M2 are also listed in Table 1. The bigger the PSNR value, the better the de-noising performance. It can be seen from Table 1 that the PSNR values of procedure M2 are greater than that of method M1 for the two images, which shows the better restoration performance of M2 over M1. Hence, the results in Figs 2 and 3, and Table 1 can show that our proposed method M2 can improve the visual quality of the restored images and PSNR significantly better than M1. The optimal experimental values of the parameters of our technique M2 (*β*_1_, *β*_2_, *α*_1_, *α*_2_), for the two images “Moon” and “Rose” are (0.003, 0.05, 0.40, 1.38) and (0.007, 0.01, 0.37, 1.41), respectively. In this example, we also set △*x* = △*y* = 10, and *dt* = 0.0002.

**Table 1:** Comparison of models M1 and M2 in terms of PSNR.

Name	Size	PSNR	
		M1	M2
Moon	300^2^	25.31	26.23
Rose	300^2^	29.23	30.30
Synthetic1	300^2^	24.93	26.01
Synthetic2	300^2^	27.09	27.96
Synthetic3	300^2^	28.25	29.22
Synthetic4	300^2^	25.73	26.60
Lena	256^2^	23.52	24.39

**Fig 4:**
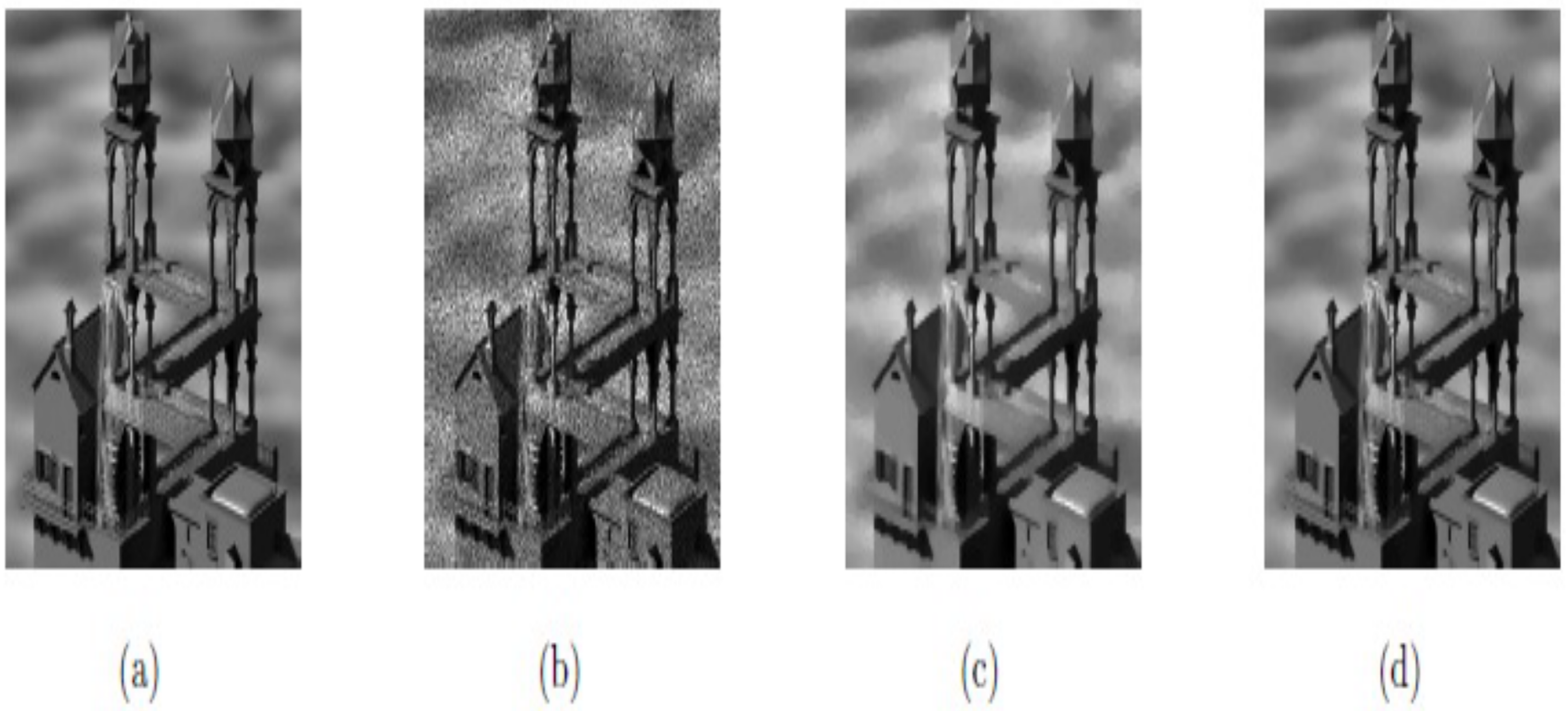
Reconstructed results on synthetic image Synthetic1; (a) Original image; (b) Noisy image with *σ*^2^ = 0.1; (c) Restored image by model M1; (d) Restored image by model M2.

**Fig 5:**
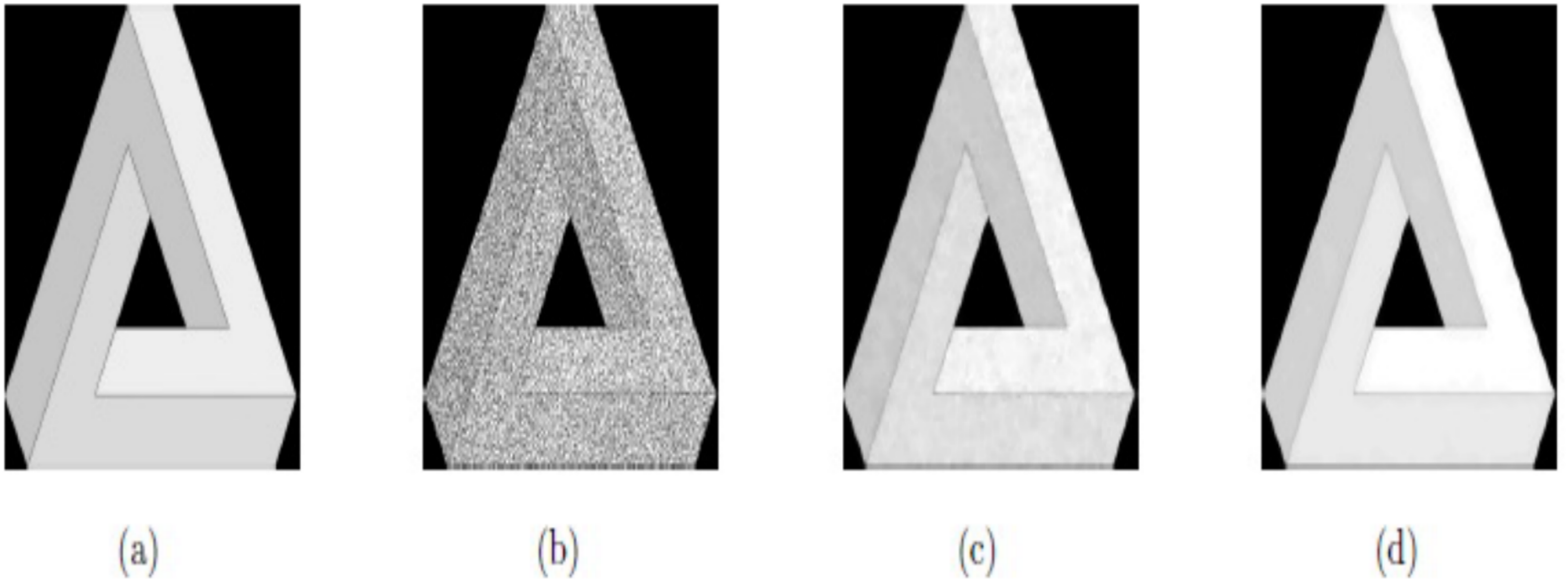
Restored results on synthetic image Synthetic2; (a) Original image; (b) Noisy image with *σ*^2^ = 0.1; (c) De-noised image by model M1; (d) De-noised image by model M2.

**Fig 6:**
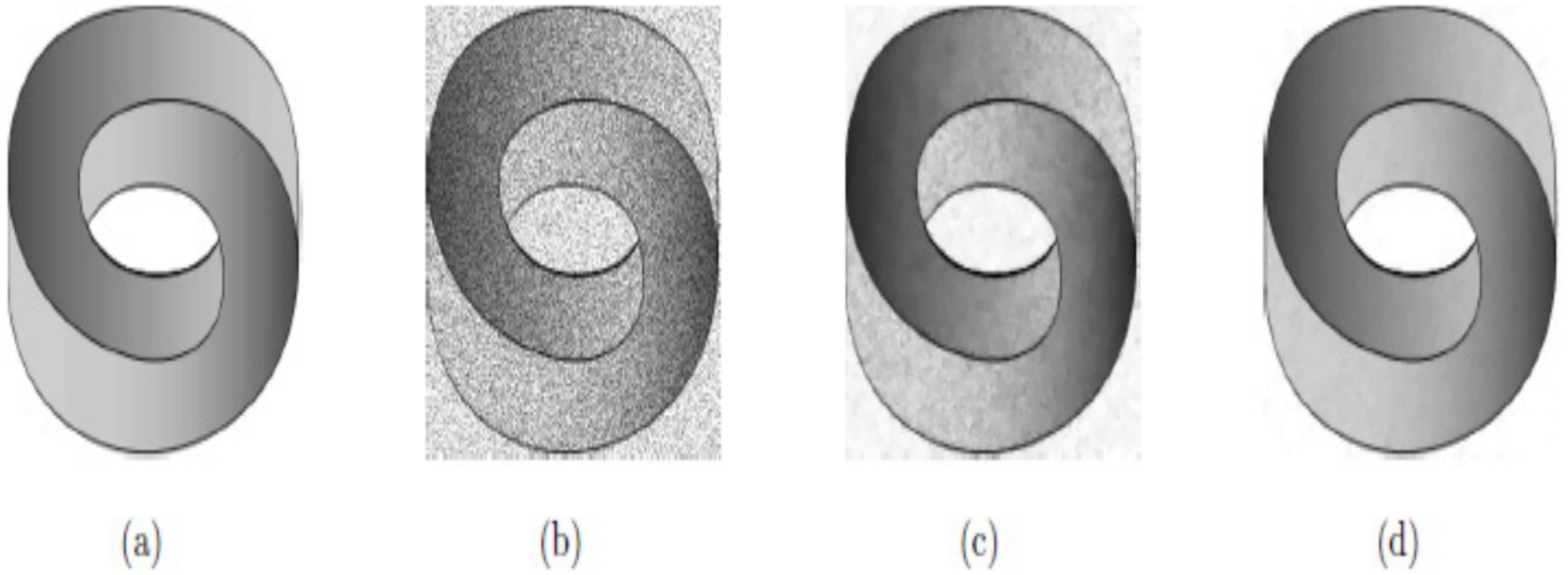
De-noised results on synthetic image Synthetic3; (a) Original image; (b) Noisy image with *σ*^2^ = 0.1; (c) Restored image by model M1; (d) Restored image by model M2.

**Fig 7:**
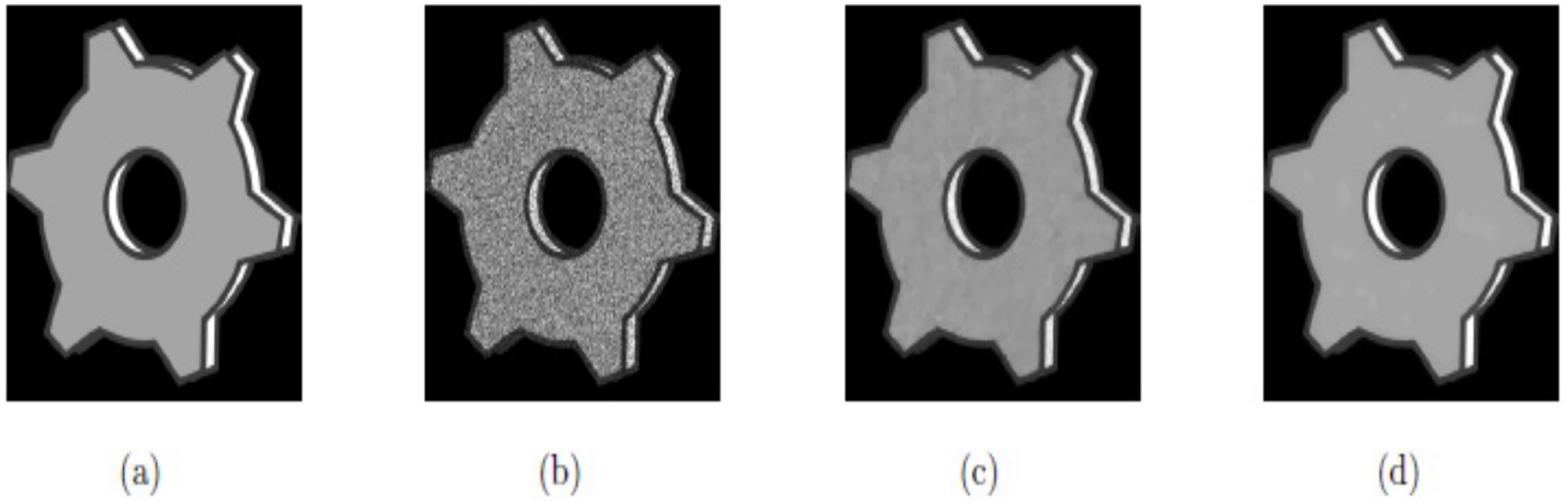
Reconstructed results on synthetic image Synthetic4; (a) Original image; (b) Noisy image with *σ*^2^ = 0.1; (c) Restored image by model M1; (d) Restored image by model M2.

**Example 2**:

In this example, the techniques M1 and M2 are tested with the synthetic images, “SynImage1”, “SynImage2”, “SynImage3”, and “SynImage4”, each of which contain speckle noise with the noise variance *σ*^2^ = 0.1. These data are shown in Figs 4, 5, 6 and 7, respectively. In these Figs and Table 1, we can observe that the restoration results using Euler’s elastica and curvature-based technique M2 are better than the TV-based technique M1 regarding visual quality and PSNR values. The best experimental optimal values of the parameters of our technique M2 (*β*_1_, *β*_2_, *α*_1_, *α*_2_) with the images “SynImage1”, “SynImage2”, “Synthetic3”, and “Synthetic4” were (0.001, 0.06, 0.34, 1.56), (0.0.004, 0.03, 0.36, 1.53), (0.002, 0.05, 0.33, 1.54), and (0.004, 0.04, 0.38, 1.30), respectively. In this example, again, we set △*x* = △*y* = 10, and *dt* = 0.0002.

**Fig 8:**
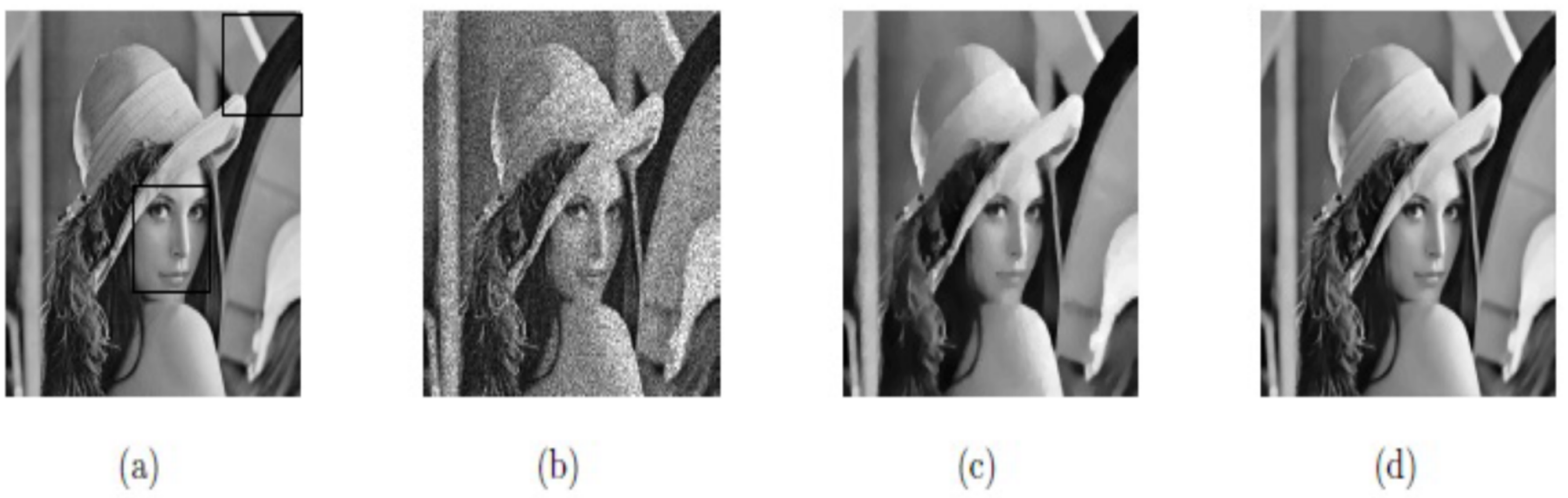
Restored results on real image Lena (a) Original image; (b) Noisy image with *σ*^2^ = 0.05; (c) Obtained image by model M1; (d) Obtained image by model M2.

**Fig 9:**
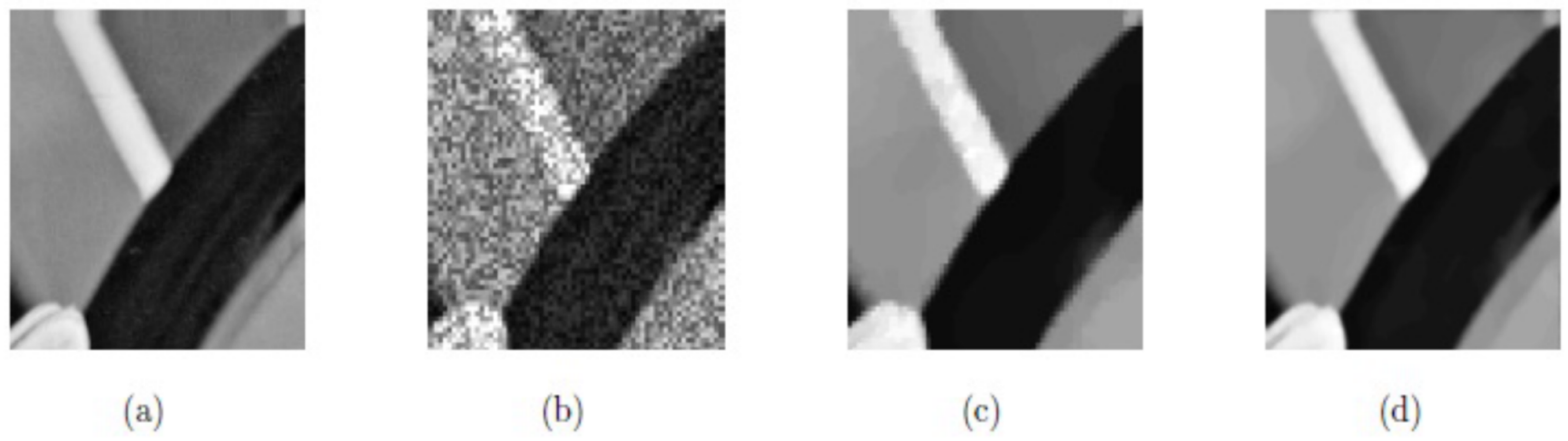
Lena with the region of interest; (a) True image; (b) Noisy image; (c) De-noised with model M1; (d) De-noised with model M2.

**Fig 10:**
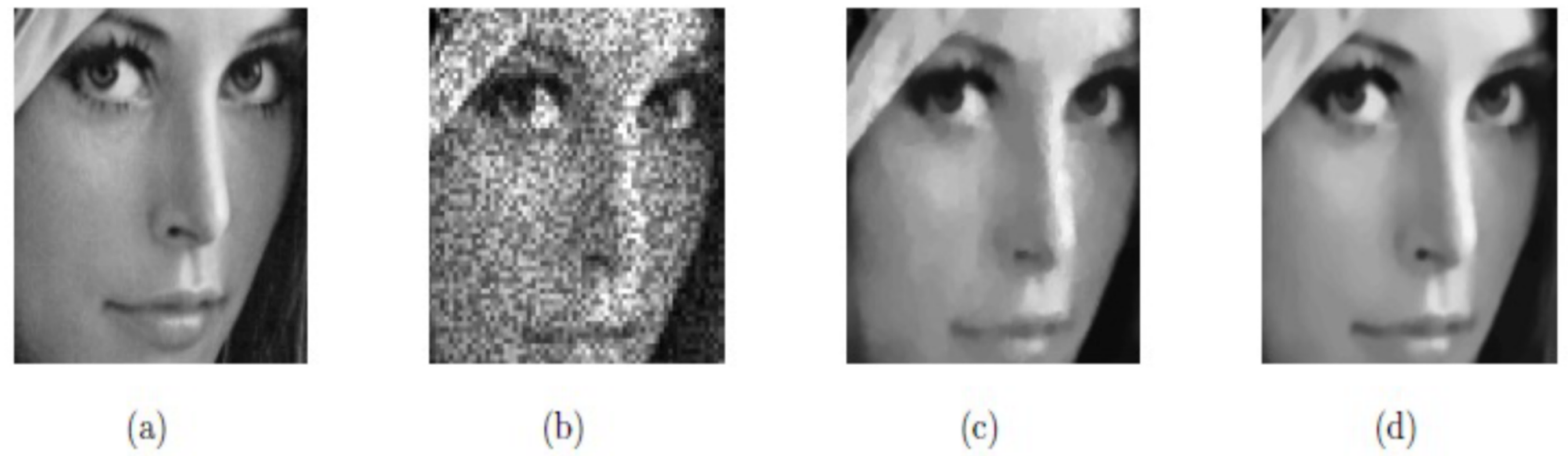
Lena with the region of interest; (a) Original image; (b) Noisy image; (c) Restored image by model M1; (d) Restored image by model M2.

**Example 3**:

Here, the image size of “Lena”, 256^2^, is taken as the test image. The main logic of our proposed model is to use Euler’s elastic curvature and Weberized TV-norm to recover both jumps and smooth signals accurately. In Figs 8, 9 and 10, and Table 1, we can observe that M2 yields better image restoration results than M1 since it preserves edges and minimizes the staircase effect. Fig 8(a) indicates the two rectangular regions of interest. Figs 9 and 10, respectively, show them zoomed in to firmly demonstrate our Euler’s elastica curvature and Weberized TV-based model M2 corresponding to TV-based model M1. The restoration results indicate that our model M2 displays better recovery results (shown in Figs 9(d) and 10(d)) compared with model M1 (shown in Figs 9(c) and 10(c)) since it preserves edges while minimizing the staircase effect as well.

**Fig 11:**
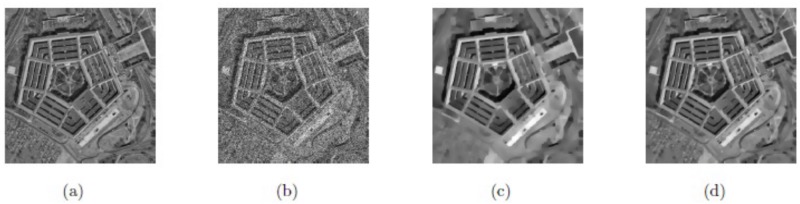
Reconstructed results on synthetic texture image; (a) Original image; (b) Noisy image; (c) De-noised image by model M1; (d) De-noised image by model M2.

**Example 4**:

In this example, a texture image of size 256^2^ is taken as the test image. Again, Fig 11 illustrates that our model M2 preserves the texture regions more than model M1, which shows the better restoration performance of our model M2. This can be seen in Fig 11(c) and 11(d), respectively.

**Fig 12:**
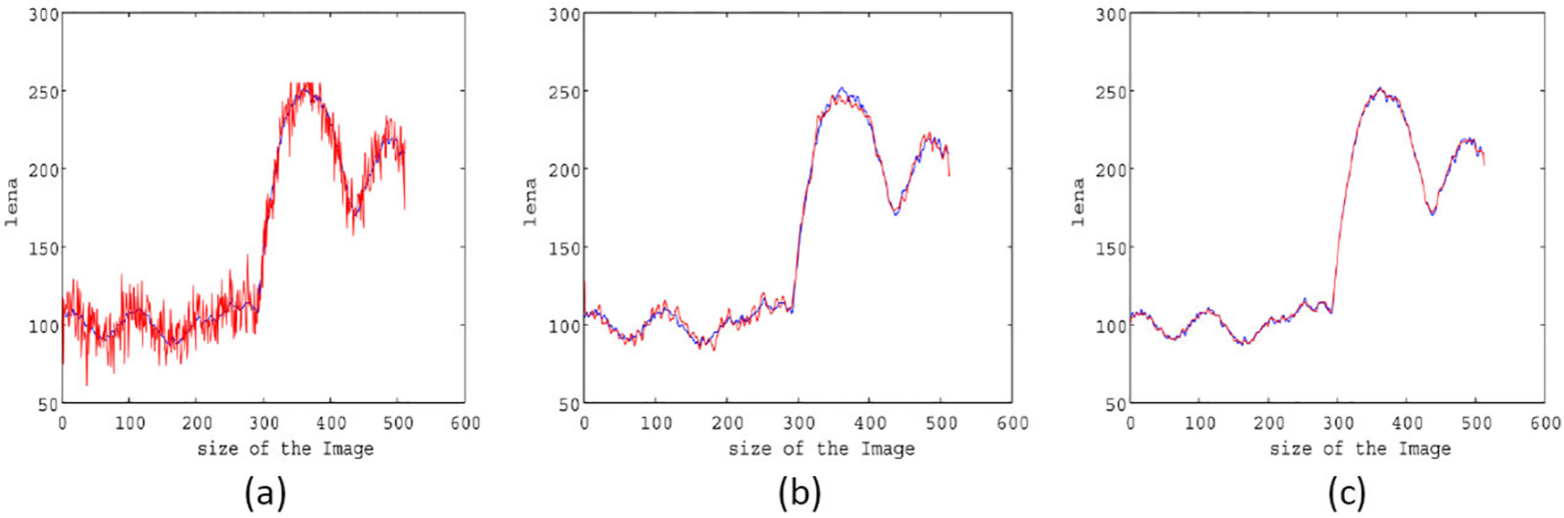
The 62*^nd^* line compression of original image with noisy image, restored image by model M1 and restored image by method M2 of the “Lena;” (a) Original and noisy image lines; (b) Original and restored by method M1 image lines; (c) Original and restored by method M2 image lines. The blue line is the original image, and the red line is the restored image.

**Example 5**:

In this example, the homogeneity is checked, and loss (or preservation) is examined for the two techniques M1 and M2 while being applied to “Lena”. For this purpose, different lines of the original image are compared to noisy and restored images that are shown in Fig 12. It is clear that the line restored by proposed method M2 (shown in Fig 12(c)) is far better than what is acquired utilizing method M1 that is presented in Fig 12(b).

**Fig 13:**
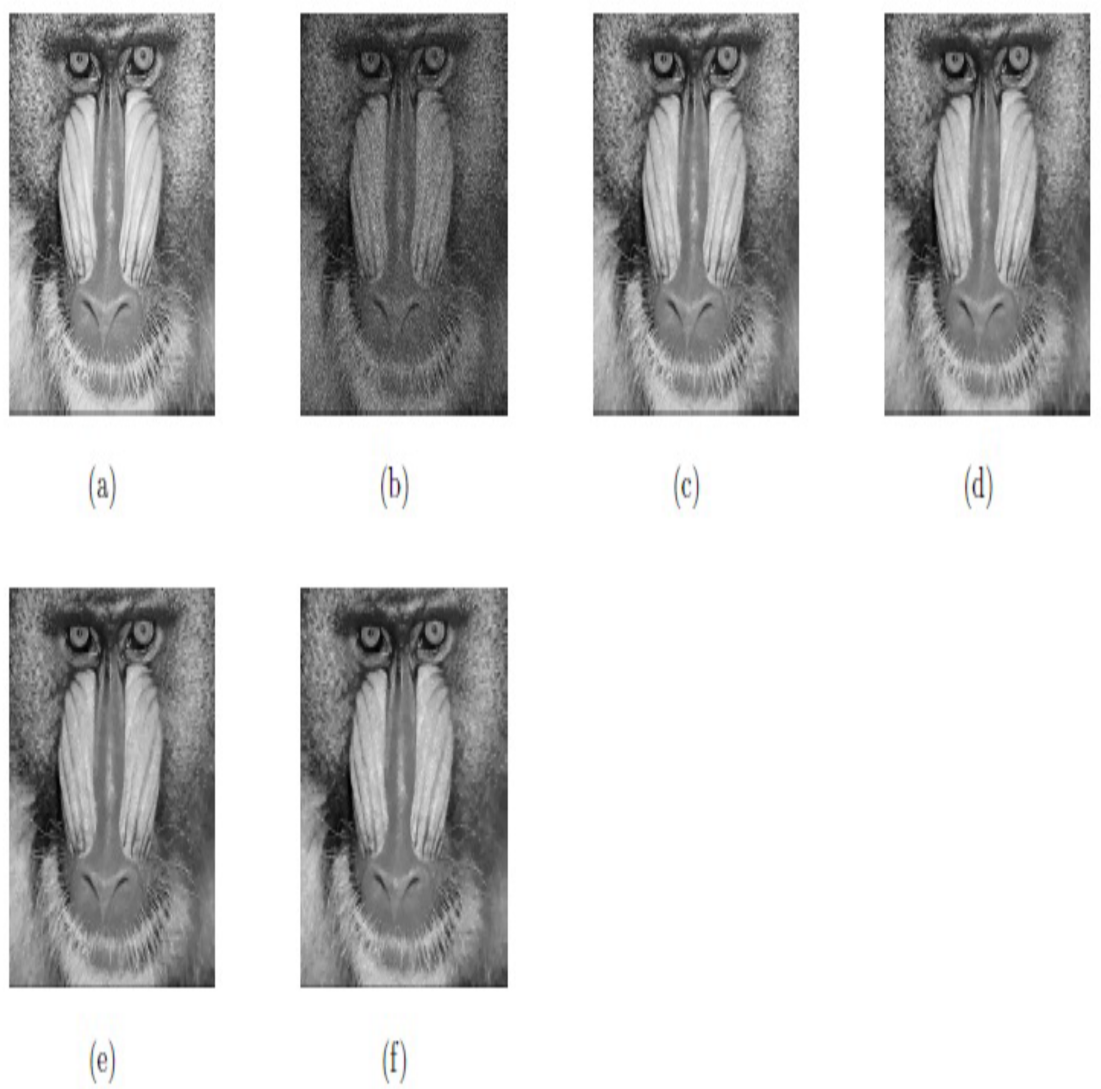
Experimental results on Baboon; (a) Original image; (b) Baboon image corrupted with multiplicative noise with *σ*^2^ = 0.1; (c) Obtained image by optimal value of *dt* = 0.0002; (d) Obtained image by decreased *dt* = 0.00002; (e) Obtained image by increased *dt* = 0.002; (f) Obtained image by increased *dt* = 0.02.

**Example 6**:

In this example, we test our proposed model M2 for different values of time step *dt* for a real image for the same values of parameters (*β*_1_, *β*_2_, *α*_1_, *α*_2_). The values of these parameters for this example are selected as (0.007, 0.093, 0.23, 1.29). So, from the Fig 13 and Table 2, we can notice that different values of time step *dt* affect the image restoration quality (PSNR) by our proposed model M2.

**Table 2:** Comparison of the image quantity (PSNR values) for different values (increase and decrease) in time step *dt* with the optimal value of time step *dt* of the proposed method M2 for real image.

Image	Size	Time step *dt*	PSNR
Baboon	300^2^	(Optimal value) *dt* = 0.0002	27.43
		(Decreased value) *dt* = 0.00002	27.23
		(Increased value) *dt* = 0.002	26.69
		(Increased value) *dt* = 0.02	25.93

Therefore, it is reasonable to conclude that Euler’s elastica curvature-based model M2 is best in the sense that it has piecewise smooth intensities, has sharp edges, and minimizes the staircase effect better than model M1.

## 7 Comparison with other methods

### 7.1 Multiplicative noise removal based on the linear alternating direction method for a hybrid variational method (M3)

Yu Hao et. al proposed a new hybrid variational model and method (in 2017) [59] based on variable splitting for multiplicative removal as follows;

$$\begin{array}{c} \underset{v,\xi }{\text{min}}\;E\left(v,\xi \right)=\int_{\Omega }|\nabla v|dx+\frac{\lambda }{2}\int_{\Omega }|\nabla \xi -v{|}^{2}dx+\alpha \int_{\Omega }|\nabla \xi |dx+\beta \int_{\Omega }(f_{0}exp\left(-\xi \right)+\xi )dx, \end{array}$$ (54)

where *ξ* = *log*(*f*) and *v* is a vector field of the image *ξ*. Also λ > 0, *α* > 0, and *β* > 0 are the regularizer parameters. Here, *f*_0_ is the observed image and *f* is the original image.

The alternating direction method has been used to solve Eq (54), which can be written into the following two minimization subproblems:

$$\begin{array}{c} v^{k+1}=arg\;\underset{v}{\text{min}}\int_{\Omega }|\nabla v|dx+\frac{\lambda }{2}\int_{\Omega }|\nabla \xi -v{|}^{2}dx, \end{array}$$ (55)

$$\begin{array}{c} \xi^{k+1}=arg\;\underset{\xi }{\text{min}}\frac{\lambda }{2}\int_{\Omega }|\nabla \xi -v^{k+1}{|}^{2}dx+\alpha \int_{\Omega }|\nabla \xi |dx+\beta \int_{\Omega }(f_{0}exp\left(-\xi \right)+\xi )dx. \end{array}$$ (56)

For the solution of the subproblem (55), the authors used the Chambolle’s dual method which is given as follows;

$$\begin{array}{c} v^{k+1}=\nabla \xi^{k+1}-\frac{1}{\lambda }divq, \end{array}$$ (57)

where $q=(\begin{array}{cc} q_{11} & q_{12}\\ q_{21} & q_{22} \end{array})$, $\nabla \xi^{k}={\left(\xi_{x}^{k},\xi_{y}^{k}\right)}^{T},$ *divq* = (*divp*_1_, *divp*_2_), *p*_1_ = (*q*_11_, *q*_12_), *p*_2_ = (*q*_21_, *q*_22_), $\xi_{x}^{k},\xi_{y}^{k}$ represent first order forward and backward differences, respectively. Then, the authors solved *p*_1_ and *p*_2_ by fixed point iteration, which are given as under.

$$\begin{array}{c} p_{1}^{k,l}=\frac{p_{1}^{k,l-1}+\tau (\nabla \left(divp_{1}^{k,l-1}\right)-\lambda \xi_{x}^{k})}{1+\tau \;|\nabla \left(divp_{1}^{k,l-1}\right)-\lambda \xi_{x}^{k}|}, \end{array}$$ (58)

$$\begin{array}{c} p_{2}^{k,l}=\frac{p_{2}^{k,l-1}+\tau (\nabla \left(divp_{2}^{k,l-1}\right)-\lambda \xi_{y}^{k})}{1+\tau \;|\nabla \left(divp_{2}^{k,l-1}\right)-\lambda \xi_{y}^{k}|}, \end{array}$$ (59)

where, $p_{1}^{k,0}=p_{2}^{k,0}=0,$ and *l* = 1.

For the solution of the subproblem (56), the authors used Bregman method by letting *η* = ∇*ξ*, which leads to the following unconstrained problem.

$$\begin{array}{c} \underset{\eta ,\xi }{\text{min}}\frac{\lambda }{2}\int_{\Omega }|\eta -v^{k+1}{|}^{2}dx+\frac{\mu }{2}∥\eta -\nabla \xi {∥}_{2}^{2}+\alpha ∥\eta {∥}_{1}+\beta \int_{\Omega }(f_{0}exp\left(-\xi \right)+\xi )dx, \end{array}$$ (60)

where *μ* > 0 is a penalty parameter. The split Bregman algorithm is defend as under;

$$\begin{array}{c} \xi^{k+1}=arg\;\underset{\xi }{\text{min}}\;\beta \int_{\Omega }(f_{0}exp\left(-\xi \right)+\xi )dx+\frac{\mu }{2}∥\eta^{k}-\nabla \xi +b^{k}{∥}_{2}^{2}. \end{array}$$ (61)

$$\begin{array}{c} \eta^{k+1}=arg\;\underset{\eta }{\text{min}}\frac{\lambda }{2}\int_{\Omega }|\eta -v^{k+1}{|}^{2}dx+\frac{\mu }{2}∥\eta -\nabla \xi^{k+1}+b^{k}{∥}_{2}^{2}+\alpha ∥\eta {∥}_{1}\\ =arg\;\underset{\eta }{\text{min}}\;\alpha ∥\eta {∥}_{1}+\frac{\lambda +\mu }{2}∥\eta -\frac{1}{\lambda +\mu }[\lambda v^{k+1}+\mu (\nabla \xi^{k+1}-b^{k})]{∥}_{2}^{2}\\ =\frac{1}{\lambda +\mu }T((\lambda v^{k+1}+\mu \left(\nabla \xi^{k+1}-b^{k}\right)),\alpha ), \end{array}$$ (62)

where

$$\begin{array}{c} b^{k+1}=b^{k}+\eta^{k+1}-\nabla \xi^{k+1}, \end{array}$$ (63)

and *T* represents the thresholding operator defined by

$$\begin{array}{c} T\left(x,\gamma \right)=\frac{x}{\left|x\right|}max\left\{|x|-\gamma ,0\right\}\;\gamma >0. \end{array}$$ (64)

The solution of the first term of Eq (62) is given as under:

$$\begin{array}{c} \xi^{k+1}=arg\;\underset{\xi }{\text{min}}\langle \beta \left(1-f_{0}exp\left(-\xi \right)\right)-\mu div\left(\eta^{k}+b^{k}-\nabla \xi^{k}\right),\xi -\xi^{k}\rangle +\frac{1}{2\sigma }∥\xi -\xi^{k}{∥}_{2}^{2}. \end{array}$$ (65)

The closest solution is obtained as follow.

$$\begin{array}{c} \xi^{k+1}=\xi^{k}-\sigma [\beta \left(1-f_{0}exp\left(-\xi \right)\right)-\mu div\left(\nabla \xi^{k}-\eta^{k}+b^{k}\right)]. \end{array}$$ (66)

**Algorithm 1**: Algorithm for method M3

1. Initialization: *v*^0^ = 0, *ξ*^0^ = *logf*_0_, *η*^0^ = 0, and *b*^0^ = 0.

2. Compute *v*^*k*+1^ by (57).

3. Compute *ξ*^*k*+1^ by (66).

4. Compute *η*^*k*+1^ by the second formula of (62).

5. Compute *η*^*k*+1^ by (63).

6. Until the stop condition is satisfied, *f*^*k*+1^ = *exp*(*ξ*^*k*+1^).

For, more information, see [59].

**Fig 14:**
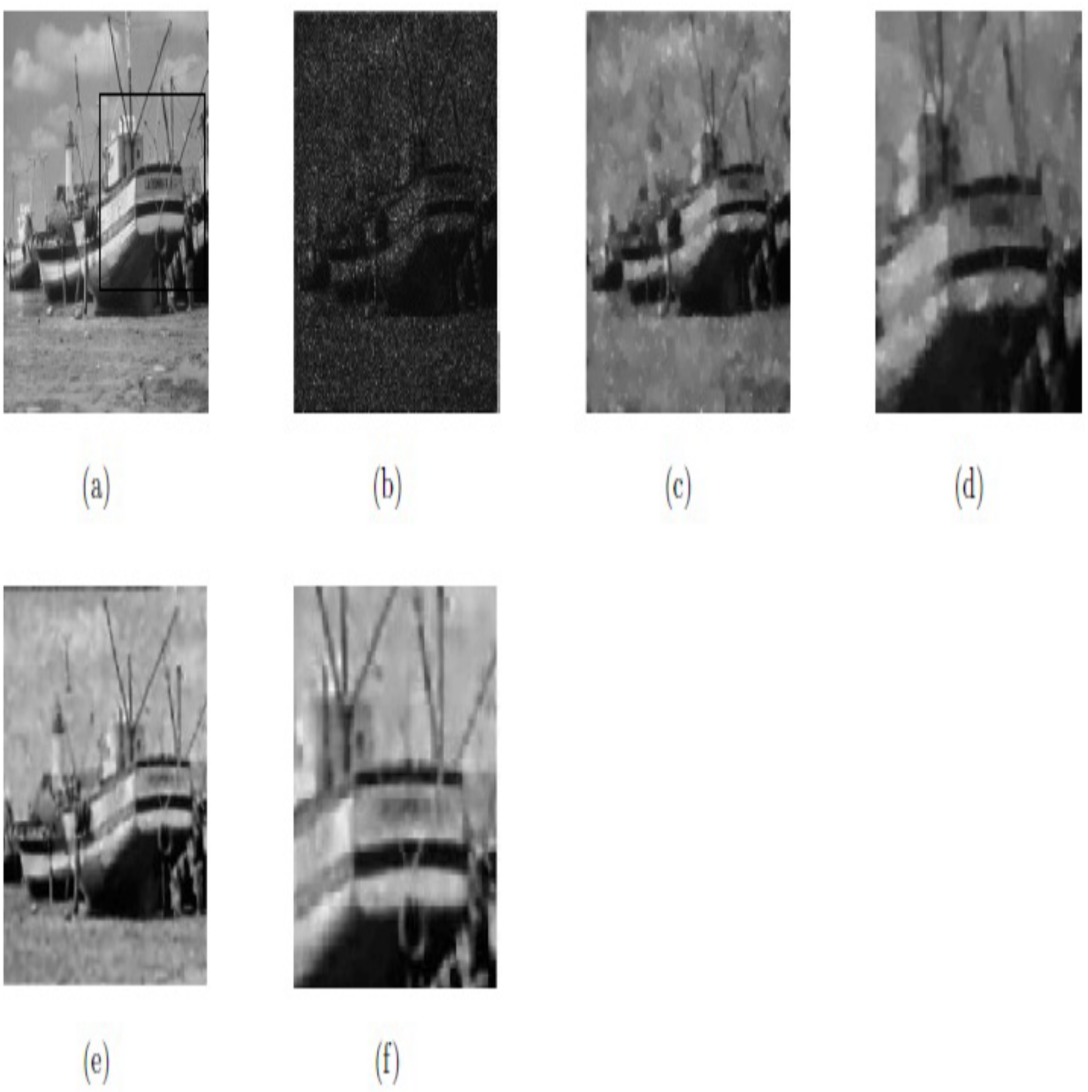
Resultant results on Boat; (a) Original image; (b) Noisy image with multiplicative noise *L* = 3; (c) De-noised image by method M3; (d) Zoomed-in part of the de-noised image by method M3; (e) De-noised image by our method M2; (f) Zoomed-in part of the denoised image by our method M2.

**Fig 15:**
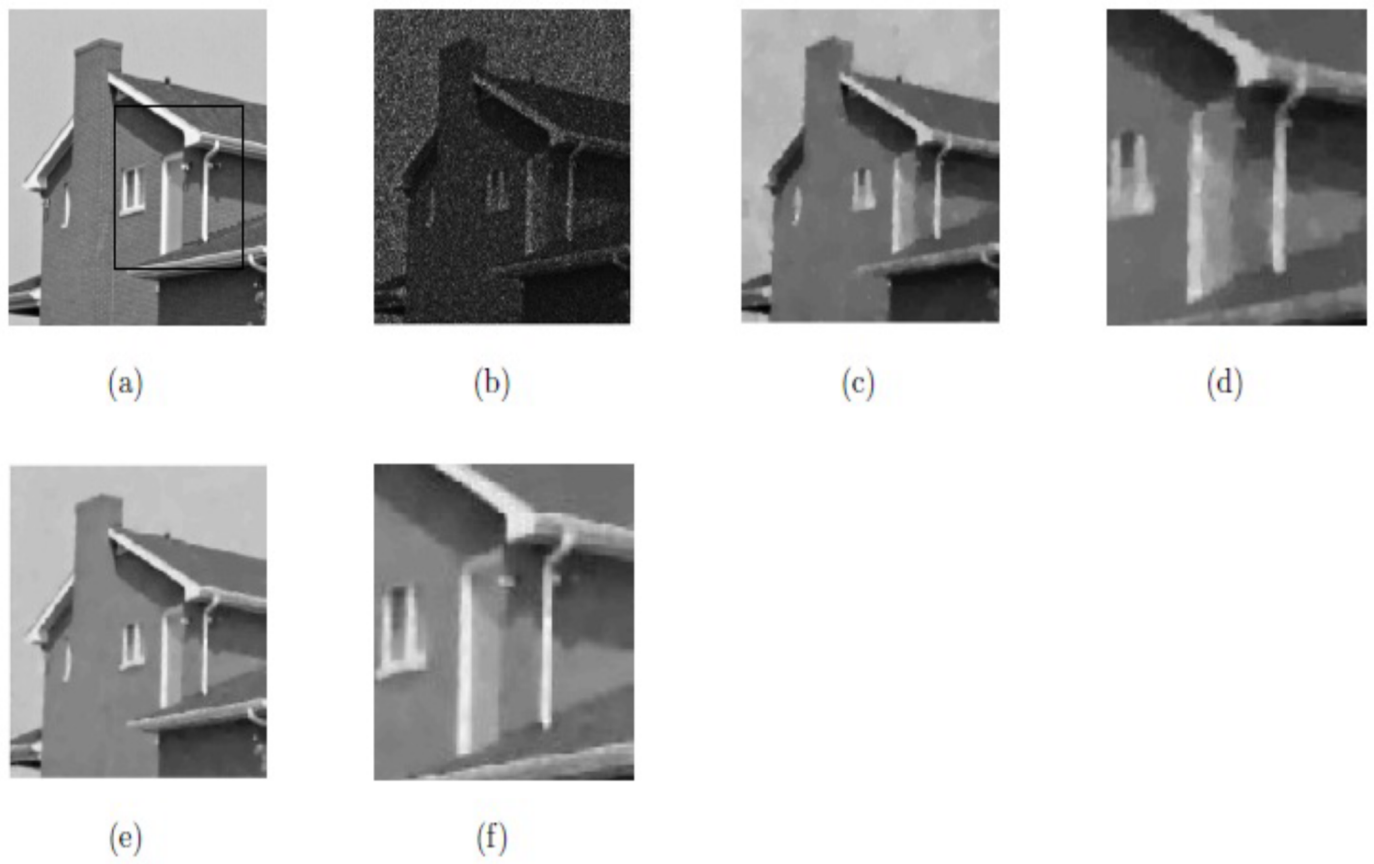
Resultant results on House; (a) Original image; (b) Noisy image with multiplicative noise *L* = 5; (c) Obtained image by method M3; (d) Zoomed-in part of the obtained image by method M3; (e) Obtained image by our method M2; (f) Zoomed-in part of the obtained image by our method M2.

In this subsection, we have compared the two models, i.e., method M3 and proposed method M2 for image restoration for the same images with the same size and noise variance along with same parameters values as selected in [59]. We can see that the results obtained by our proposed method M2 are outstanding in the visual quality of restoration (PSNR), eliminating the staircase effect and preserving the textures. These obtained results are shown in Figs 14 and 15, and Table 3, respectively. The optimal values of parameters for our proposed method M2 (*β*_1_, *β*_2_, *α*_1_, *α*_2_) for the two images “Boat” and “House” are (0.01, 0.008, 0.23, 1.05), and (0.01, 0.0062, 0.17, 1.04), respectively. In this case, we choose △*x* = △*y* = 10, and *dt* = 0.0002.

**Table 3:** Comparison of method M3 and our method M2 regarding of PSNR.

Image	Size	Method M3	Our method M2
		PSNR	PSNR
Boat	256^2^	22.48	23.93
House	256^2^	25.47	26.61

### 7.2 Multiplicative noise removal combining a total variation regularizer and a nonconvex regularizer (M4)

Y. Han et. al proposed a new variational model (in 2014) [60] and hence some good restoration results have been obtained. The minimization functional for this model is given as follow:

$$\begin{array}{c} \underset{u,b}{\text{min}}\;E(u,b)=\lambda \int_{\Omega }(u+ze^{-u})dx+\int_{\Omega }(\epsilon +\left(1-\epsilon \right)b^{2})\left|Du\right|+\left(1-\epsilon \right)\int_{\Omega }(1-\frac{b}{\sqrt{\alpha }})dx, \end{array}$$ (67)

where the second term is called the weighted total variation term, function $b:\Omega \to [0,\sqrt{\alpha }],$ *ϵ* is the positive parameter such that 0 < *ϵ* < 1. Also the second term ∫_*Ω*_(*ϵ* + (1 − *ϵ*)*b*^2^)|*Du*|, is finite. However, *u* ∈ *BV*(*Ω*) doest not mean that ∫_*Ω*_ *b*^2^|*Du*| is also definitely finite. Hence, once ∫_*Ω*_ *b*^2^|*Du*| is infinite, ∫_*Ω*_(*ϵ* + (1 − *ϵ*)*b*^2^)|*Du*| can not be decomposed into the sum of *ϵ*∫_*Ω*_|*Du*| and (1 + *ϵ*)∫_*Ω*_|*Du*|. The authors used an alternating iteration process directly of (67) which includes two minimization problems as follows.

First by fixing *b*, they solve

$$\begin{array}{c} \underset{u}{\text{min}}\{J(u)=\lambda \int_{\Omega }(u+ze^{-u})dx+\int_{\Omega }(\epsilon +\left(1-\epsilon \right)b^{2})\left|Du\right|\}. \end{array}$$ (68)

Second, with *u* fixed, they solve

$$\begin{array}{c} \underset{b}{\text{min}}\{F(b)=\int_{\Omega }(\epsilon +\left(1-\epsilon \right)b^{2})\left|Du\right|+\left(1-\epsilon \right)\int_{\Omega }(1-\frac{b}{\sqrt{\alpha }})dx\}. \end{array}$$ (69)

The given algorithm shows the alternating iteration process for the task of removing multiplicative noise. The only challenging issue is to minimize the functional (70) in Algorithm 2, or to minimize the general functional in the minimization problem (68). For this purpose, the authors convert the minimization functional (68) equivalently for constructing more efficient solver, which is given as under.

**Algorithm 2**: Algorithm for model M4

Initialization process:

Let *u*_0_ = *logz*, $b_{0}=\sqrt{\alpha },$ set the iteration index *k* = 0, and given parameter λ, *ϵ*(0 < *ϵ* < 1), *α*, *σ*.

Iteration process:

$$\begin{array}{c} u_{k+1}=arg\;\underset{u}{\text{min}}\{\lambda \int_{\Omega }(u+ze^{-u})dx+\int_{\Omega }(\epsilon +\left(1-\epsilon \right)b_{k}^{2})\left|Du\right|\}; \end{array}$$ (70)

$$\begin{array}{c} {\tilde{u}}_{k+1}=\frac{1}{\sigma^{2}}u_{k+1}⊗\beta \left(\frac{x}{\sigma }\right); \end{array}$$ (71)

$$\begin{array}{c} b_{k+1}=\frac{\sqrt{\alpha }}{1+\alpha |\nabla {\tilde{u}}_{k+1}|}. \end{array}$$ (72)

Determination process:

If *u*_*k*+1_ and *u*_*k*_ satisfy ∫_Ω_(*u*_*k*+1_ − *u*_*k*_)*dx* < |Ω|*τ* (*τ* shows fixed threshold), then stop the whole process and output $\tilde{u}=u_{k+1}$ and $\tilde{b}=b_{k+1}.$ Otherwise, set *k* = *k* + 1 and return to the iteration process.

$$\begin{array}{c} \underset{u}{\text{min}}\{\lambda \int_{\Omega }(u+ze^{-u})dx+\int_{\Omega }(\epsilon +\left(1-\epsilon \right)b^{2})\left|Dv\right|\}, \end{array}$$ (73)

s.t *u* = *v*.

The authors then used the alternating direction of multipliers(ADMM) method to solve the constrained minimization problem (73) which is shown in the following iteration process in Algorithm 3.

**Algorithm 3**: Algorithm for model Minimization functional (73)

Initialization process:

Given parameters λ and *μ*, set the iteration index *n* = 0, and let *u*_0_ = *v*_0_, *d*_*n*_ = 0;

Iteration process:

$$\begin{array}{c} u_{n+1}=arg\;\underset{u}{\text{min}}\{\lambda \int_{\Omega }(u+ze^{-u})dx+\frac{\mu }{2}\int_{\Omega }{\left(u-v_{n}+d_{n}\right)}^{2}dx\}; \end{array}$$ (74)

$$\begin{array}{c} v_{n+1}=arg\;\underset{v}{\text{min}}\{\frac{\mu }{2}\int_{\Omega }{\left(u_{n+1}-v+d_{n}\right)}^{2}dx+\int_{\Omega }(\epsilon +\left(1-\epsilon \right)b^{2})\left|Dv\right|\}; \end{array}$$ (75)

$$\begin{array}{c} d_{n+1}=d_{n}+\left(u_{n+1}-v_{n+1}\right) \end{array}$$ (76)

Determination process:

Once the sequence {*u*_*n*_} and {*v*_*n*_} converge when *n* → ∞, stop the algorithm, otherwise, set *n* = *n* + 1 and return to the iteration process.

The Euler Lagrange equation of the functional in the minimization problem (74) is given as under;

$$\begin{array}{c} \lambda \left(1-ze^{-u}\right)+\mu \left(u-v_{n}+d_{n}\right)=0. \end{array}$$ (77)

The solution of above Eq (77) is obtained by using the few times Newton iterations. The solution to the (75) is obtained by the following Chambolle’s projection algorithm;

$$\begin{array}{c} v_{n+1}=u_{n+1}+d_{n}-\frac{\lambda }{\mu }divq, \end{array}$$ (78)

where *q*: Ω → *R* × *R* shows the vector function, div notation shows the divergence operator. The function *q* is the limit of the sequence *q*_*m*_ (the index *m* differs from the outer loop index *n*) generated from the following fixed point iteration: given an initial *q*_0_ and a time-step *ϱ*, the following iterative scheme is used:

$$\begin{array}{c} q_{n+1}=\frac{q_{m}+\varrho \nabla (divq_{m}-\left(\mu /\lambda \right)\left(u_{n+1}+d_{n}\right))}{1+\left(\frac{\varrho }{\epsilon }+\left(1-\epsilon \right)b^{2}\right)\left|divq_{m}-\left(\mu /\lambda \right)\left(u_{n+1}+d_{n}\right)\right|}. \end{array}$$ (79)

According to the authors, due to the nonconvex nature of minimization problem (69) it is hard to prove the global convergence about the whole algorithm in Algorithm 2. For, further details, see [60].

**Fig 16:**
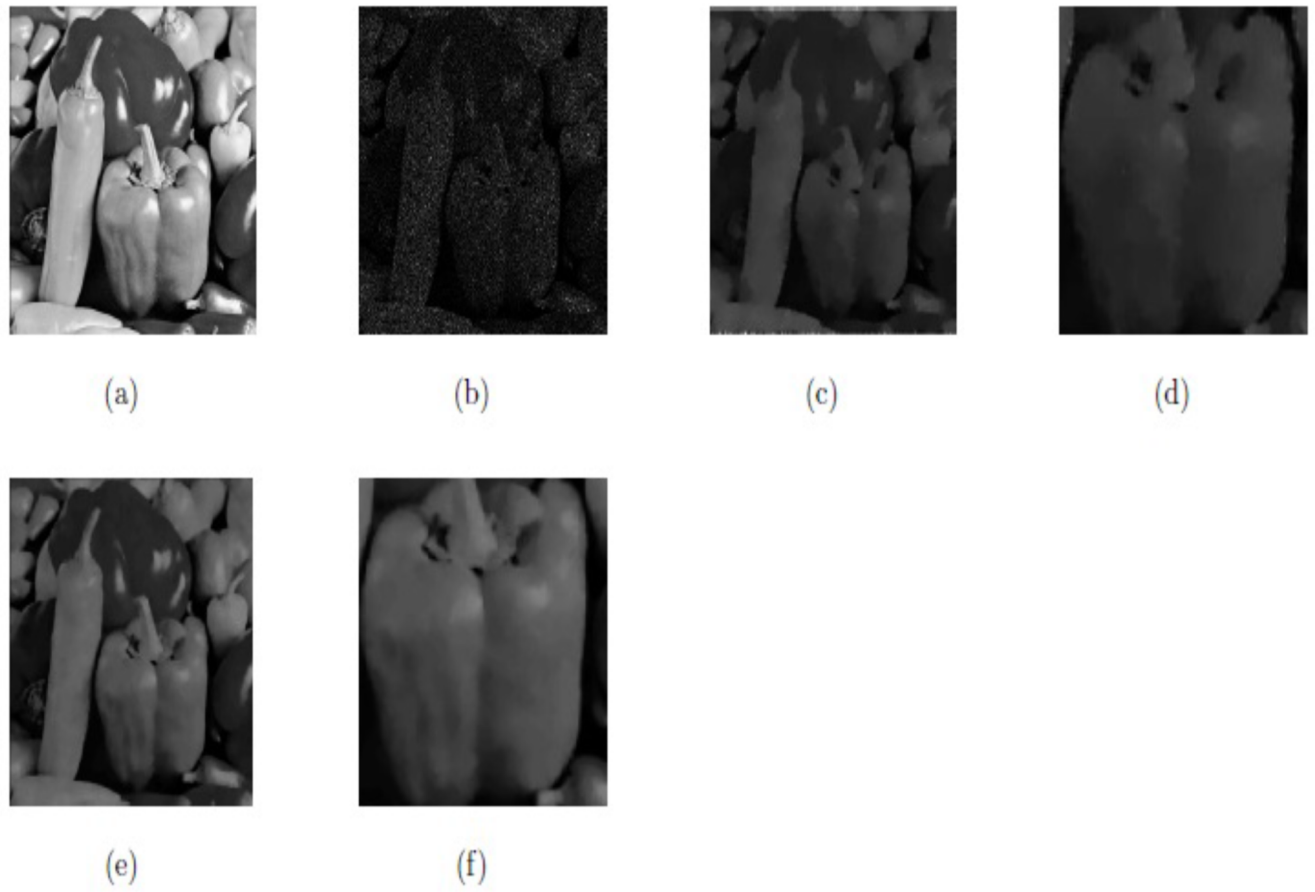
Resultant results on Peppers; (a) Original image; (b) Noisy image with multiplicative noise *L* = 3; (c) Recorded image by method M4; (d) Zoomed-in part of the recovered image by method M4; (e) Recorded image by our method M2; (f) Zoomed-in part of the recovered image by our method M2.

**Fig 17:**
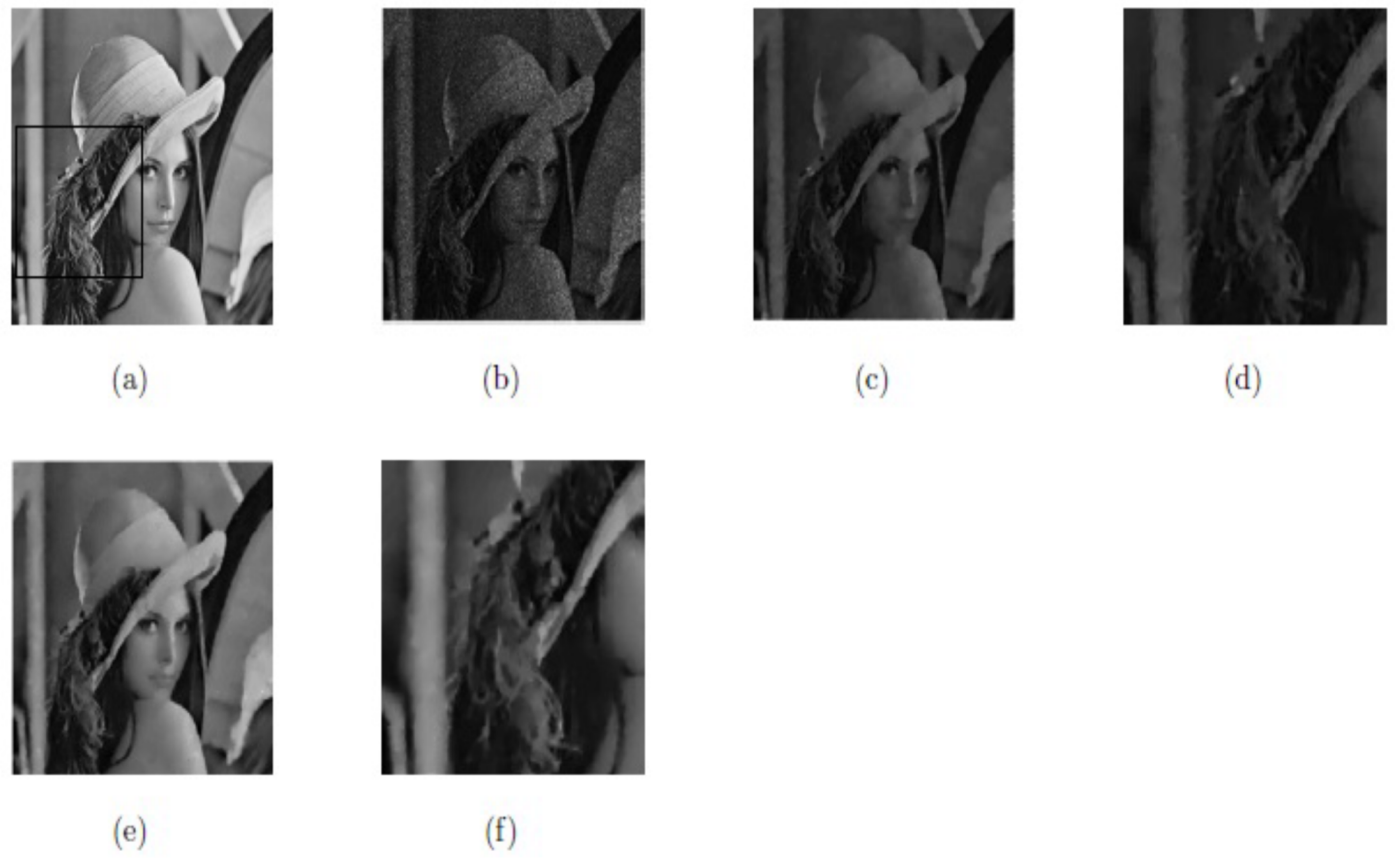
De-noised results on Lena; (a) Original image; (b) noisy image with multiplicative noise *L* = 15; (c) Reconstructed image by method M4; (d) Zoomed-in part of the reconstructed image by method M4; (e) Reconstructed image by our method M2; (f) Zoomed-in part of the reconstructed image by method M2.

Here, the model M4 that is proposed in [60] is compared with our proposed method M2 for the same images having the same size and noise variances and same parameters values that have been selected in [60]. Again, from Figs 16 and 17, and Table 4 we can observe that our proposed technique M2 has better performance in the visual quality of restoration (SNR), reducing the staircase effect and preserving the textures compared to the model M4. The values of the parameters selected for our proposed model M2 (*β*_1_, *β*_2_, *α*_1_, *α*_2_) for the two images “Peppers” and “Lena” are (0.01, 0.0085, 0.24, 1.05), and (0.02, 0.005, 0.20, 1.02), respectively. In this case, we select △*x* = △*y* = 10, and *dt* = 0.0002.

**Table 4:** Comparison of method M4 and our method M2 regarding of PSNR.

Image	Size	Method M3	Our method M2
		PSNR	PSNR
Peppers	256^2^	22.62	23.79
Lena	256^2^	26.99	28.05

## 8 Sensitivity analysis of parameters

To briefly comment on the choice of the various parameters used in the algorithm of our model M2, it must be noted that *β*_1_, *β*_2_, *α*_1_ and *α*_2_ are more complicated to choose according to our experience. The difficulty of tuning these parameters is that they not only depend on the noise level, but also on the type of images. However, their optimal values are adjusted and tuned according to the noise variance and the image. It was observed that the ranges of values allowed are: *β*_1_ ∈ [0.0025, 0.0097], *β*_2_ ∈ [0.0082, 0.074], *α*_1_ ∈ [0.29, 0.50] and *α*_2_ ∈ [1.22, 1.61] for natural and synthetic images according to the noise variance *σ*^2^ = 0.1. Using, these ranges, better restoration results could be achieved with improved PSNR results. Results are presented in Tables 5 and 6.

**Table 5:** The PSNR value of the restored image “Moon” with optimal values of *β*_1_, *β*_2_, *α*_1_ and *α*_2_ is 26.23. Parameter sensitivity analysis of our proposed model M2 by percentage increased in values of the parameters *β*_1_, *β*_2_, *α*_1_ and *α*_2_, with the resultant percentage increase or decrease in PSNR of the de-noised image of size (300^2^).

Image	40% (↑)					70% (↑)				
	*β*_1_	*β*_2_	*α*_1_	*α*_2_	PSNR	*β*_1_	*β*_2_	*α*_1_	*α*_2_	PSNR
Moon	0.0042	0.0700	0.5600	1.9320	2.38(↓)	0.0051	0.0850	0.6800	2.3460	3.62(↓)

**Table 6:** The PSNR value of the restored image “Moon” with optimal values of *β*_1_, *β*_2_, *α*_1_ and *α*_2_ is 26.23. Parameter sensitivity analysis for our proposed model M2 by percentage decreased in values of the parameters *β*_1_, *β*_2_, *α*_1_ and *α*_2_, with the resultant percentage increase or decrease in PSNR of the de-noised image of size (300^2^).

Image	40% (↓)					70% (↓)				
	*β*_1_	*β*_2_	*α*_1_	*α*_2_	PSNR	*β*_1_	*β*_2_	*α*_1_	*α*_2_	PSNR
Moon	0.0018	0.0300	0.2400	0.8280	3.15(↓)	9e-4	0.0150	0.1200	0.4140	4.21(↓)

For brevity, the following notations are utilized in these tables.

1. (⋅)%*increase*− ↑, and (⋅)%*decrease*− ↓
2. For example (0.50) ↓ stands for 0.50% decrease in PSNR
3. (0.30) ↑ stands for 0.30% increase in PSNR

## 9 Conclusion

In this paper, a new high-order model was introduced using Euler’s elastica curvature combined with the total variation regularization for image restoration with speckle noise. The implicit gradient descent scheme was exploited for solving nonlinear PDE arisen from the minimization of the proposed functional. The experimental results demonstrated that the proposed model improves PSNR and can preserve edges, textures and minimize the staircase effect compared with existing PDE-based models. The sensitivity analysis of parameters was also discussed in details. Our model has also been compared with other variational PDE-based models for image restoration when the noise variance is large.

In our model the resulting Euler-Lagrange equation has fourth order derivatives and is also anisotropic and highly nonlinear, and thus the conventional algorithms struggle to solve it efficiently due to the stability restriction. This problem is under intense study and results will be reported in the subsequent paper.

## Appendix A

To derive the Euler-Lagrange Eq (26), we use the first variation or optimality condition [28, 56, 57] of the functional (26), that is

$$\begin{array}{c} \frac{d}{d\epsilon }(E(g+\epsilon \phi ){|}_{\epsilon \to 0}=\frac{d}{d\epsilon }[(\frac{\beta_{1}g+\beta_{2}}{g})\{\alpha_{1}E_{1}(g+\epsilon \phi )+\alpha_{2}E_{2}(g+\epsilon \phi )+E_{3}(g+\epsilon \phi )\}]{|}_{\epsilon \to 0}=0. \end{array}$$ (80)

It means that we split the energy into three parts ie *E*_1_(*g*), *E*_2_(*g*) and *E*_3_(*g*) respectively.

$$\begin{array}{c} E_{1}(g+\epsilon \phi )=\int_{\Omega }|\nabla (g+\epsilon \phi )|dxdy. \end{array}$$ (81)

$$\begin{array}{c} E_{2}(g+\epsilon \phi )=\int_{\Omega }|{\left(\nabla \cdot \frac{\nabla g}{\left|\nabla g\right|}\right)}^{2}|\nabla (g+\epsilon \phi )dxdy. \end{array}$$ (82)

$$\begin{array}{c} E_{3}(g+\epsilon \phi )=\int_{\Omega }log(g+\epsilon \phi )+\frac{f}{g(g+\epsilon \phi )}dxdy. \end{array}$$ (83)

Now to compute *E*_1_ from Eq (81):

$$\begin{array}{c} \frac{d}{d\epsilon }(E_{1}(g+\epsilon \phi )){|}_{\epsilon \to 0}=\frac{d}{d\epsilon }\int_{\Omega }|\nabla (g+\epsilon \phi )|dxdy{|}_{\epsilon \to 0}, \end{array}$$ (84)

$$\begin{array}{c} \frac{d}{d\epsilon }(E_{1}(g+\epsilon \phi )){|}_{\epsilon \to 0}=\frac{d}{d\epsilon }\int_{\Omega }\frac{\nabla (g+\epsilon \phi )}{\left|\nabla (g+\epsilon \phi )\right|}\cdot \nabla (\phi )dxdy{|}_{\epsilon \to 0}, \end{array}$$ (85)

$$\begin{array}{c} \frac{d}{d\epsilon }(E_{1}(g+\epsilon \phi )){|}_{\epsilon \to 0}=\frac{d}{d\epsilon }\int_{\Omega }\frac{\nabla g}{\left|\nabla (g\right|}\cdot \nabla \left(\phi \right)dxdy, \end{array}$$ (86)

$$\begin{array}{c} \frac{d}{d\epsilon }(E_{1}(g+\epsilon \phi )){|}_{\epsilon \to 0}=-\frac{d}{d\epsilon }\int_{\Omega }\nabla \cdot \frac{\nabla g}{\left|\nabla g\right|}\phi dxdy+\int_{\Omega }\frac{\nabla g}{\left|\nabla g\right|}\cdot nds. \end{array}$$ (87)

But at the ∂Ω ∇*g* ⋅ *n* = 0. So, the above Eq (87) can be written as

$$\begin{array}{c} \frac{d}{d\epsilon }(E_{1}(g+\epsilon \phi )){|}_{\epsilon \to 0}=-\int_{\Omega }\nabla \cdot \frac{\nabla g}{\left|\nabla g\right|}\phi dxdy. \end{array}$$ (88)

To compute *E*_2_ from Eq (82):

$$\begin{array}{c} \frac{d}{d\epsilon }(E_{2}(g+\epsilon \phi )){|}_{\epsilon \to 0}=\frac{d}{d\epsilon }\int_{\Omega }{\left(\nabla \cdot \frac{\nabla (g+\epsilon \phi )}{\left|\nabla (g+\epsilon \phi )\right|}\right)}^{2}|\nabla (g+\epsilon \phi )|dxdy{|}_{\epsilon \to 0}, \end{array}$$ (89)

$$\begin{array}{ccc} \frac{d}{d\epsilon }(E_{2}(g+\epsilon \phi )){|}_{\epsilon \to 0} & = & \int_{\Omega }\frac{d}{d\epsilon }{\left(\nabla \cdot \frac{\nabla (g+\epsilon \phi )}{\left|\nabla (g+\epsilon \phi )\right|}\right)}^{2}{|}_{\epsilon \to 0}|\nabla g|dxdy\\ & + & \int_{\Omega }\frac{d}{d\epsilon }{\left(\nabla \cdot \frac{\nabla g}{\left|\nabla g\right|}\right)}^{2}\frac{d}{d\epsilon }(\nabla g+\epsilon \phi )dxdy{|}_{\epsilon \to 0}. \end{array}$$ (90)

From above Eq (90), we have

$$\begin{array}{c} \frac{d}{d\epsilon }(E_{2}(g+\epsilon \phi )){|}_{\epsilon \to 0}=A+B, \end{array}$$ (91)

where

$$\begin{array}{c} A=\int_{\Omega }\frac{d}{d\epsilon }{\left(\nabla \cdot \frac{\nabla (g+\epsilon \phi )}{\left|\nabla (g+\epsilon \phi )\right|}\right)}^{2}{|}_{\epsilon \to 0}|\nabla g|dxdy. \end{array}$$ (92)

and

$$\begin{array}{c} B=\int_{\Omega }\frac{d}{d\epsilon }{\left(\nabla \cdot \frac{\nabla g}{\left|\nabla g\right|}\right)}^{2}\frac{d}{d\epsilon }(\nabla g+\epsilon \phi )dxdy{|}_{\epsilon \to 0}. \end{array}$$ (93)

From (92)

$$\begin{array}{c} A=\int_{\Omega }\frac{d}{d\epsilon }{\left(\nabla \cdot \frac{\nabla (g+\epsilon \phi )}{\left|\nabla g+\epsilon \phi )\right|}\right)}^{2}{|}_{\epsilon \to 0}|\nabla g|dxdy=\int_{\Omega }2\kappa |\nabla g|\frac{d}{d\epsilon }(\nabla \cdot \frac{\nabla (g+\epsilon \phi )}{\left|\nabla (g+\epsilon \phi )\right|})dxdy, \end{array}$$ (94)

$$\begin{array}{c} A=\int_{\Omega }\frac{d}{d\epsilon }{\left(\nabla \cdot \frac{\nabla (g+\epsilon \phi )}{\left|\nabla (g+\epsilon \phi )\right|}\right)}^{2}{|}_{\epsilon \to 0}|\nabla g|dxdy=\int_{\Omega }2\kappa |\nabla g|\nabla \cdot [(\nabla \cdot \frac{\nabla (\phi )}{\left|\nabla g\right|}-\frac{\nabla g}{|\nabla g{|}^{3}}\nabla g\cdot \nabla \phi )]dxdy. \end{array}$$ (95)

Here, we obtained the term $\vec{n}⊕\vec{n}=(\frac{\nabla \phi \cdot \nabla g}{{\left|\nabla g\right|}^{2}})\nabla g$, is the orthogonal projection into the normal direction which salifies $\vec{n}⊕\vec{n}+t⊕t=I$ with *t* ⊕ *t* is orthogonal projection and *I* is the identity projection. So from (95) we have

$$\begin{array}{c} A=\int_{\Omega }\frac{d}{d\epsilon }{\left(\nabla \cdot \frac{\nabla (g+\epsilon \phi )}{\left|\nabla (g+\epsilon \phi )\right|}\right)}^{2}{|}_{\epsilon \to 0}|\nabla g|dxdy=\int_{\Omega }2\kappa |\nabla g|[\frac{1}{\left|\nabla g\right|}\{t⊕t\}\nabla \phi ]dxdy, \end{array}$$ (96)

$$\begin{array}{ccc} A=\int_{\Omega }\frac{d}{d\epsilon }{\left(\nabla \cdot \frac{\nabla (g+\epsilon \phi )}{\left|\nabla (g+\epsilon \phi )\right|}\right)}^{2}{|}_{\epsilon \to 0}|\nabla g|dxdy & = & -\int_{\Omega }[(\nabla \cdot (2\kappa |\nabla g|))(\frac{1}{\left|\nabla g\right|}\{t⊕t\}\nabla \phi )]dxdy\\ & + & \int_{\partial \Omega }2\kappa n\cdot \{t⊕t\}\nabla \phi ds. \end{array}$$ (97)

But at the ∂Ω

$$\begin{array}{c} \int_{\partial \Omega }2\kappa n\cdot \{t⊕t\}\nabla \phi ds=\int_{\partial \Omega }2\kappa n\cdot \{n\times n\}\nabla \phi ds=\int_{\partial \Omega }2\kappa n\cdot \nabla \phi =0. \end{array}$$ (98)

Then, the (97) becomes

$$\begin{array}{c} A=\int_{\Omega }\frac{d}{d\epsilon }{\left(\nabla \cdot \frac{\nabla (g+\epsilon \phi )}{\left|\nabla (g+\epsilon \phi )\right|}\right)}^{2}{|}_{\epsilon \to 0}|\nabla g|dxdy=-\int_{\Omega }[\nabla \cdot (2\kappa |\nabla g|)(\frac{1}{\left|\nabla g\right|}\{t⊕t\}\nabla \phi )]dxdy. \end{array}$$ (99)

As {*t* ⊕ *t*} is symmetric. The Eq (99) can be written as

$$\begin{array}{c} A=\int_{\Omega }\frac{d}{d\epsilon }{\left(\nabla \cdot \frac{\nabla (g+\epsilon \phi )}{\left|\nabla (g+\epsilon \phi )\right|}\right)}^{2}{|}_{\epsilon \to 0}|\nabla g|dxdy=-\int_{\Omega }\frac{1}{\left|\nabla g\right|}\{t⊕t\}\nabla (2\kappa |\nabla g|)\cdot \nabla \phi dxdy,by\;parts. \end{array}$$ (100)

$$\begin{array}{cc} A=\int_{\Omega }\frac{d}{d\epsilon }{\left(\nabla \cdot \frac{\nabla (g+\epsilon \phi )}{\left|\nabla (g+\epsilon \phi )\right|}\right)}^{2}{|}_{\epsilon \to 0}|\nabla g|dxdy & =\int_{\Omega }(\nabla \cdot \{t⊕t\}\frac{1}{\left|\nabla g\right|}\nabla (2\kappa |\nabla g|)\cdot \phi dxdy\\ & -\int_{\partial \Omega }(\{t⊕t\}\frac{1}{\left|\nabla g\right|}(2\kappa |\nabla g|))\cdot nds \end{array}$$ (101)

But at the ∂Ω $(\{t⊕t\}\frac{1}{\left|\nabla g\right|}(2\kappa |\nabla g|))\cdot n=0$ as (2*κ*(|∇*g*|)) ⋅ *n* = 0. So, from (101) we have

$$\begin{array}{c} A=\int_{\Omega }\frac{d}{d\epsilon }{\left(\nabla \cdot \frac{\nabla (g+\epsilon \phi )}{\left|\nabla (g+\epsilon \phi )\right|}\right)}^{2}{|}_{\epsilon \to 0}|\nabla g|dxdy=\int_{\Omega }(\nabla \cdot \{t⊕t\}\frac{1}{\left|\nabla g\right|}\nabla (2\kappa |\nabla g|))\cdot \phi dxdy. \end{array}$$ (102)

From (93) we have

$$\begin{array}{c} B=\int_{\Omega }\frac{d}{d\epsilon }{\left(\nabla \cdot \frac{\nabla g}{\left|\nabla g\right|}\right)}^{2}\frac{d}{d\epsilon }\nabla (g+\epsilon \phi ){|}_{\epsilon \to 0}dxdy=\int_{\Omega }\kappa^{2}\frac{\nabla g}{\left|\nabla g\right|}\cdot \nabla \phi dxdy, \end{array}$$ (103)

$$\begin{array}{c} B=\int_{\Omega }\frac{d}{d\epsilon }{\left(\nabla \cdot \frac{\nabla g}{\left|\nabla g\right|}\right)}^{2}\frac{d}{d\epsilon }\nabla (g+\epsilon \phi ){|}_{\epsilon \to 0}dxdy=-\int_{\Omega }\nabla \cdot (\kappa^{2}\frac{\nabla g}{\left|\nabla g\right|})\phi dxdy+\int_{\partial \Omega }\kappa^{2}\frac{\nabla g}{\left|\nabla g\right|}\cdot nds. \end{array}$$ (104)

Since, on the ∂Ω ∇*g* ⋅ *n* = 0. So (104) becomes

$$\begin{array}{c} B=\int_{\Omega }\frac{d}{d\epsilon }{\left(\nabla \cdot \frac{\nabla g}{\left|\nabla g\right|}\right)}^{2}\frac{d}{d\epsilon }\nabla (g+\epsilon \phi ){|}_{\epsilon \to 0}dxdy=-\int_{\Omega }\nabla \cdot (\kappa^{2}\frac{\nabla g}{\left|\nabla g\right|})\phi dxdy. \end{array}$$ (105)

Now, combining Eqs (91), (102) and (105), we get

$$\begin{array}{c} \frac{d}{d\epsilon }(E_{2}(g+\epsilon \phi )){|}_{\epsilon \to 0}=\int_{\Omega }(\nabla \cdot \{t⊕t\}\frac{1}{\left|\nabla g\right|}\nabla (2\kappa |\nabla g|))\cdot \phi dxdy-\int_{\Omega }\nabla \cdot (\kappa^{2}\frac{\nabla g}{\left|\nabla g\right|})\phi dxdy. \end{array}$$ (106)

To compute *E*_3_ from Eq (83):

$$\begin{array}{c} \frac{d}{d\epsilon }\left(E_{3}\left(g+\epsilon \phi \right)\right){|}_{\epsilon \to 0}=\frac{d}{d\epsilon }\underset{\Omega }{\int }\left(log\left(g+\epsilon \phi \right)+\frac{f}{\left(g+\epsilon \phi \right)}\right)dxdy, \end{array}$$ (107)

$$\begin{array}{c} \frac{d}{d\epsilon }(E_{3}(g+\epsilon \phi )){|}_{\epsilon \to 0}=\int_{\Omega }(\frac{1}{g}\phi -\frac{f}{g^{2}}\phi )dxdy, \end{array}$$ (108)

$$\begin{array}{c} \frac{d}{d\epsilon }(E_{3}(g+\epsilon \phi )){|}_{\epsilon \to 0}=\int_{\Omega }(\frac{g-f}{g^{2}})\phi dxdy. \end{array}$$ (109)

From (80), (88), (106) and (109) we have

$$\begin{array}{c} -(\frac{\beta_{1}g+\beta_{2}}{g})[\nabla \cdot (\alpha_{1}+\alpha_{2}\kappa^{2})\frac{\nabla g}{\left|\nabla g\right|}\phi +\alpha_{2}\{t⊕t\}\frac{1}{\left|\nabla u\right|}\nabla (2\kappa |\nabla g|)\phi ]+(\frac{g-f}{g^{2}})\phi =0, \end{array}$$ (110)

$$\begin{array}{c} -\nabla \cdot (\alpha_{1}+\alpha_{2}\kappa^{2})\frac{\nabla g}{\left|\nabla g\right|}+\alpha_{2}\{t⊕t\}\frac{1}{\left|\nabla g\right|}\nabla (2\kappa |\nabla g|)+\frac{1}{g(\beta_{1}g+\beta_{2})}(g-f)=0. \end{array}$$ (111)

Since $\vec{n}=\nabla g=(g_{x},g_{y}),t=\nabla^{⊥}g=(-g_{y},g_{x})$ and $\tilde{\lambda }=(\frac{1}{g(\beta_{1}u+\beta_{2})})$. So, Eq (111) becomes

$$\begin{array}{c} -\nabla \cdot (\alpha_{1}+\alpha_{2}\kappa^{2})\frac{\nabla g}{\left|\nabla g\right|}+\frac{2\alpha_{2}}{|\nabla g{|}^{3}}\nabla^{⊥g}(\nabla (\kappa |\nabla g|))\nabla^{⊥}+\tilde{\lambda }(g-f)=0, \end{array}$$ (112)

is the required Euler-Lagrange equation, which implies

$$\begin{array}{c} -(\nabla \cdot U)+\tilde{\lambda }(g-f)=0, \end{array}$$ (113)

where

$$\begin{array}{c} U=[(\alpha_{1}+\alpha_{2}\kappa^{2})\cdot \frac{\nabla g}{\left|\nabla g\right|}-\frac{2\alpha_{2}}{|\nabla g{|}^{3}}\nabla^{⊥}g\nabla (\kappa |\nabla g|)\nabla^{⊥}g]. \end{array}$$ (114)

Here *U* = (*U*^1^, *U*^2^), with

$$\begin{array}{c} U^{1}=(\alpha_{1}+\alpha_{2}\kappa^{2})\frac{\partial_{x}g}{\left|\nabla g\right|}-\frac{2\alpha_{2}}{|\nabla g{|}^{3}}[-\partial_{y}g\partial_{x}(\kappa |\nabla g|)+\partial_{x}g\partial_{y}(\kappa |\nabla g|)]\partial_{y}g. \end{array}$$ (115)

$$\begin{array}{c} U^{2}=(\alpha_{1}+\alpha_{2}\kappa^{2})\frac{\partial_{y}g}{\left|\nabla g\right|}-\frac{2\alpha_{2}}{|\nabla g{|}^{3}}[-\partial_{y}g\partial_{x}(\kappa |\nabla g|)+\partial_{x}g\partial_{y}(\kappa |\nabla g|)]\partial_{x}g. \end{array}$$ (116)
